# Bispecific immunotherapy based on antibodies, T-cell receptors, and aptamers: mechanisms of action, adverse effects, and future perspectives

**DOI:** 10.3389/fimmu.2025.1679092

**Published:** 2025-11-04

**Authors:** Julia A. Lopatnikova, Sergey V. Sennikov

**Affiliations:** Laboratory of Molecular Immunology, Research Institute of Fundamental and Clinical Immunology, Novosibirsk, Russia

**Keywords:** bispecific antibodies, T-cell receptor, TCR-based therapeutics, aptamers, immunotherapy, cytokine release syndrome, hematologic malignancies, solid tumor

## Abstract

Over the past decade, bispecific immunotherapeutic platforms have progressed from laboratory prototypes to multicenter clinical trials, inaugurating a new trajectory for precision oncology. This review synthesizes original studies that address the design principles, mechanisms of action, therapeutic efficacy, and limitations of three principal classes of bispecific molecules: (i) IgG-like antibodies, (ii) modified T-cell-receptor-based constructs (TCR-like and ImmTAC), and (iii) bispecific aptamers. IgG formats—including blinatumomab, teclistamab, mosunetuzumab, and tarlatamab—achieve high objective-response rates in hematologic malignancies and are increasingly demonstrating clinical activity in solid tumors. TCR-based constructs broaden the repertoire of actionable targets by recognizing intracellular antigens presented on MHC molecules, as exemplified by the approval of tebentafusp for uveal melanoma. Aptameric molecules exhibit minimal immunogenicity, rapid tissue penetration, and considerable promise as carriers for therapeutic payloads. We provide an in-depth analysis of the signaling cascades activated during T- and NK-cell redirection, immune checkpoint blockade, and direct inhibition of oncogenic receptors. Comparative evaluation of completed and ongoing clinical studies highlights recurring challenges and adverse events associated with bispecific platforms, including cytokine-release syndrome, neurotoxicity, antigenic drift, limited infiltration of densely fibrotic solid tumors, and the emergence of anti-drug antibodies. Engineering solutions under development encompass protease-activatable “masked” constructs, step-up dosing regimens, enzymatic remodeling of the extracellular matrix, and local expression of engager molecules via oncolytic viruses or adeno-associated viral vectors. Special emphasis is placed on combinatorial strategies in which bispecific agents are paired with CAR-T or γδ-T cells, PD-(L)1 inhibitors, or oncolytic viruses, thereby enhancing effector-cell infiltration and curtailing resistance. The integrated evidence indicates that continued progress in bispecific immunotherapy will depend on the incorporation of predictive molecular biomarkers, dynamic monitoring of the evolving antigenic landscape, and the standardization of biomanufacturing processes. These advances are expected to accelerate the clinical deployment of next-generation, multipurpose bispecific constructs.

## 1 Introduction

Immunotherapy occupies a central position in contemporary oncology and represents a cornerstone of personalized medicine. One of the most rapidly evolving approaches in this field is bispecific immunotherapy, which relies on molecules capable of simultaneously recognizing two distinct targets. These constructs create new opportunities for redirecting and activating immune cells by enabling the concurrent engagement of tumor and effector components, blocking key signaling pathways, and overcoming mechanisms of immune evasion (1–3). To date, multiple formats of bispecific molecules have been developed, including IgG-like antibodies, BiTE constructs, TCR-based designs, and aptamer hybrids (4, 5).

Bispecific immunotherapy has demonstrated its greatest therapeutic impact in hematologic malignancies; agents such as blinatumomab, mosunetuzumab, and teclistamab have already been incorporated into standard-of-care regimens for relapsed and refractory disease (1, 5). In recent years, the application of bispecific antibodies has expanded to solid tumors, with approvals now granted for non-small-cell lung cancer, neuroendocrine tumors, uveal melanoma, and cholangiocarcinoma (6, 7).

The principal advantage of bispecific constructs lies in their multimodal activity, which permits the simultaneous activation of immune cells and inhibition of oncogenic signaling cascades (4, 8). Nevertheless, the clinical use of these agents faces several obstacles, including the risk of cytokine-release syndrome, limited penetration into tumor tissue, antigenic drift, and engineering challenges (9, 10). Promising avenues include the development of activatable formats, multifunctional platforms, and combination regimens with immune-checkpoint inhibitors (5).

This review aims to systematize current approaches to bispecific immunotherapy, encompassing antibody-, TCR-, and aptamer-based constructs, their mechanisms of action, clinical potential, limitations, and future directions.

## 2 Biological basis and mechanisms of action of bispecific antibodies

### 2.1 Structure and principal formats of bispecific antibodies

Bispecific antibodies (BsAbs) constitute a rapidly advancing class of immunotherapeutic agents that can engage two different epitopes simultaneously. This dual specificity not only directs cytotoxic effector cells—such as T and NK lymphocytes—toward tumor targets but also blocks critical signaling pathways sustaining malignant growth and survival (2, 5). In contrast to monoclonal antibodies, which recognize a single epitope, bispecific constructs deliver multi-targeted activity, a property of particular value in the context of highly heterogeneous tumors (9).

**Figure 1:**
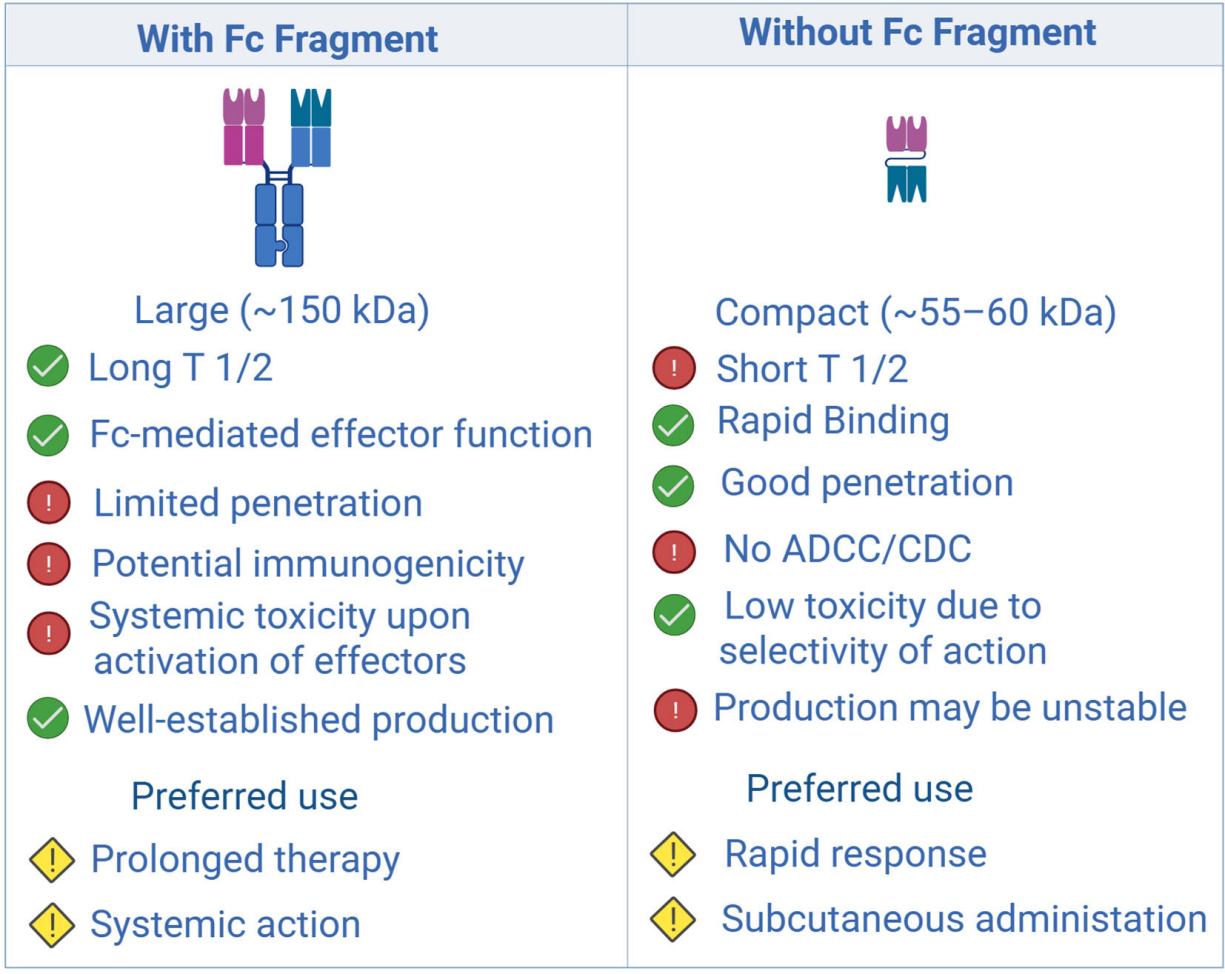
Comparison of structural, pharmacokinetic, and functional characteristics of antibodies with and without an Fc fragment: impact on therapeutic applications. Created with Biorender.com.

BsAbs can be divided into two broad classes: (i) molecules that lack an Fc domain and (ii) full-length antibodies that retain the Fc region (**Figure 1**). The first category includes BiTE, DART, and TandAb constructs, which exhibit rapid tissue diffusion and potent cytotoxicity (11–13). However, their short serum half-life—attributable to the absence of neonatal Fc-receptor (FcRn) recycling—necessitates continuous infusion or engineering modifications to extend exposure (14).

**Figure 2:**
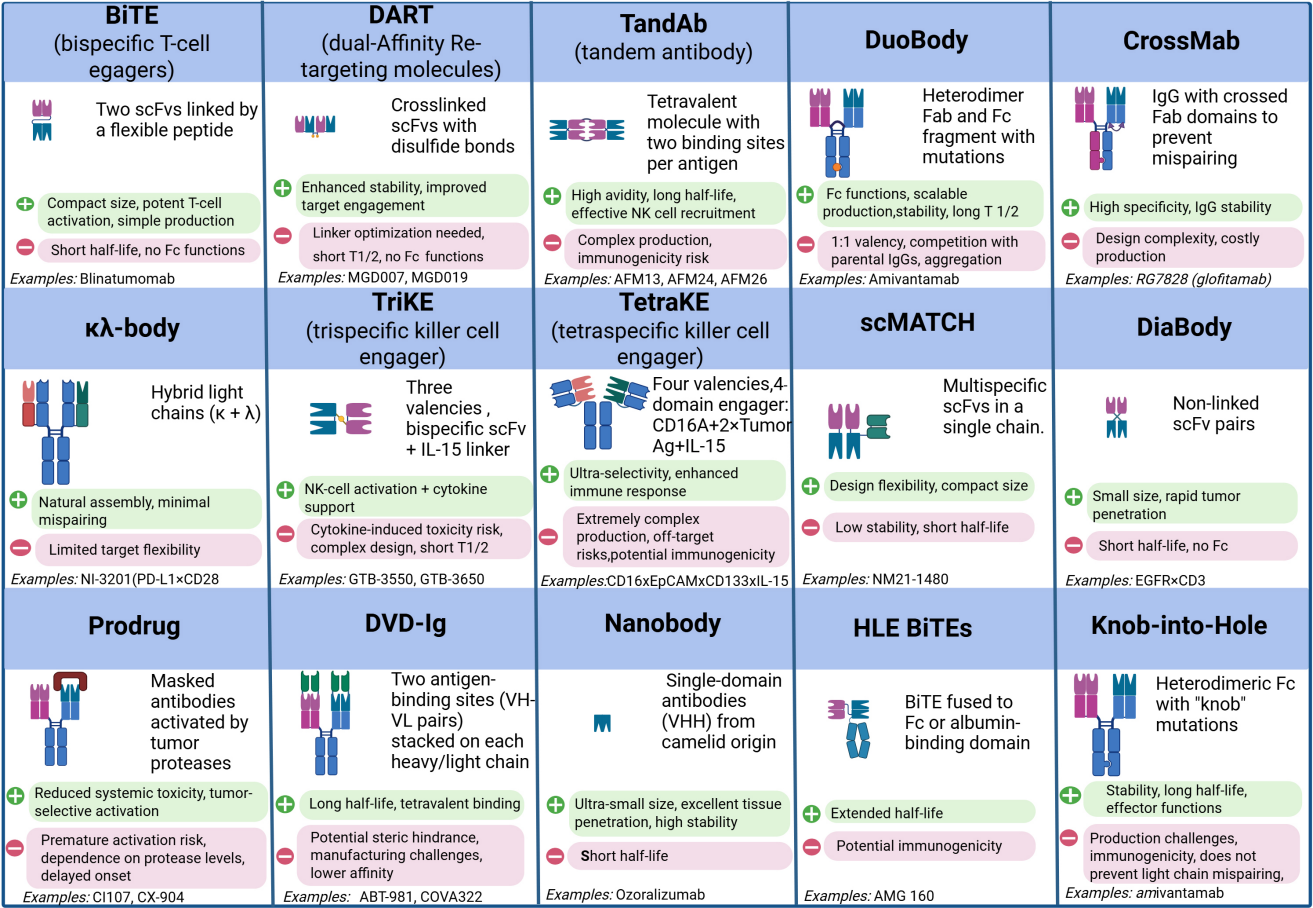
Comparative analysis of modern bispecific and multispecific antibody formats: structural design, functional properties, and therapeutic applications. Created with Biorender.com.

Full-length BsAbs, such as DuoBody, CrossMab, and κλ-body, adopt an IgG-like architecture. The presence of an Fc fragment confers improved pharmacokinetics and enables Fc-mediated effector functions, including antibody-dependent cellular cytotoxicity (ADCC) and complement-dependent cytotoxicity (CDC) (15, 16). Nevertheless, their larger size restricts penetration into solid tumors, particularly in hypoxic niches with extensive stromal remodeling (17, 18). Hybrid formats that combine a compact size with prolonged circulation—e.g., by incorporating albumin-binding domains—are under development to overcome this limitation (12, 19) (**Figure 2**).

Contemporary platforms focus on streamlining chain pairing and enhancing product homogeneity: CrossMab employs domain exchange, whereas DuoBody relies on controlled Fab-arm exchange (2, 20, 21). Multifunctional constructs such as TriKEs and tetraspecific antibodies, which concomitantly activate T and NK cells and deliver immunomodulators like IL-15, are also gaining traction (22, 23). A key priority remains the fine-tuning of antigen affinity to minimize binding to healthy tissues (24, 25). Moreover, conditionally active formats that become functional only within the tumor microenvironment—triggered by low pH or protease activity—are being investigated to enhance selectivity and reduce systemic toxicity (8, 26).

Despite significant engineering advances, challenges related to stability, aggregation, and product heterogeneity persist. Strategies to address these issues include Fc modifications, nanotechnology-based delivery systems, and sequence optimizations that facilitate expression and correct assembly (27, 28).

The diversity of formats and structural solutions not only defines the pharmacological profile of bispecific antibodies but also determines the nature of their interactions with the immune system, which underlies their distinct mechanisms of action.

### 2.2 Principal mechanisms of action of bispecific antibodies

**Figure 3:**
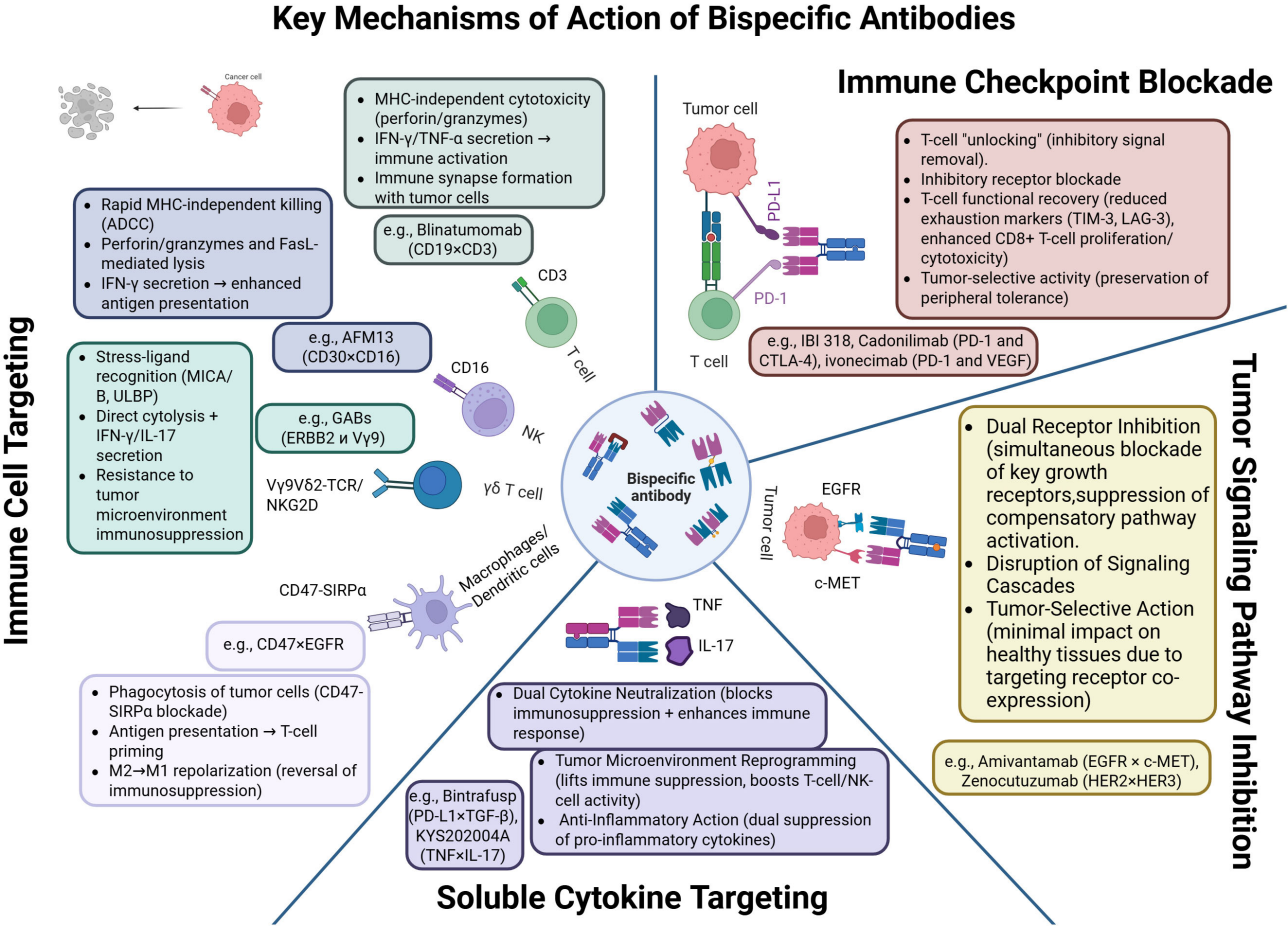
Mechanisms of action of bispecific antibodies: cytotoxicity, immune synapse formation, checkpoint blockade, and tumor microenvironment reprogramming. Created with Biorender.com.

The immunotherapeutic potential of bispecific antibodies (BsAbs) derives from their capacity to redirect cytotoxic effector cells, block immune checkpoints, and disrupt the signaling networks that sustain tumor growth and survival (**Figure 3**).

The clinically most consequential mechanism is T-cell redirection. BiTE constructs such as blinatumomab bring CD3^+^ T-lymphocytes into contact with tumor cells, forming an immune synapse that triggers perforin- and granzyme-mediated apoptosis of the target cell (5, 29). Because their activity is independent of MHC presentation and co-stimulation, these molecules have a broad therapeutic range; tuning CD3 affinity can mitigate cytokine-release syndrome (CRS) (30). Analogously, NK-cell engagers—for example AFM13 (CD30/CD16A)—activate NK cells via FcγRIIIa, inducing antibody-dependent cellular cytotoxicity (ADCC) (31). Incorporating IL-15 into trispecific formats (TriKEs) further promotes NK-cell proliferation and persistence (32). At the signaling level, BsAbs trigger phosphorylation of ZAP-70, LAT, and PLCγ1 in T cells, leading to Ca²^+^ mobilization, NFAT activation, and engagement of the MAPK/ERK pathway (33, 34); in NK cells they activate SYK and PI3K, driving granule exocytosis and synthesis of IFN-γ and TNF-α (35).

BsAbs that co-target immune checkpoints (e.g., PD-1/CTLA-4, PD-1/LAG-3, PD-1/TIGIT) enable more localized immune activation while limiting systemic hyper-stimulation. IBI318, which binds PD-1 and PD-L1, augments T-cell reactivation (36), whereas MGD019 (PD-1/CTLA-4) lowers expression of exhaustion markers such as LAG-3 and TIM-3 (37).

A further mode of action involves direct blockade of oncogenic signaling. Zenocutuzumab (HER2/HER3) inhibits the PI3K/AKT cascade in tumors harboring NRG1 fusions (38). Amivantamab (EGFR/MET) combines receptor inhibition with ADCC and has shown efficacy in non-small-cell lung cancer with EGFR exon-20 insertions (39).

BsAbs directed against soluble cytokines are also attracting interest. M7824 (bintrafusp alfa), which simultaneously blocks PD-L1 and neutralizes TGF-β, exerts synergistic modulation of the tumor microenvironment and restores T-cell activity (40). In autoimmune and inflammatory diseases, BsAbs that co-neutralize TNF-α and IL-17 are effective in psoriasis and Crohn’s disease (41). Concurrent inhibition of IL-4 and IL-13 offers a promising strategy for asthma, providing more comprehensive Th2 suppression than dupilumab (42, 43).

Collectively, bispecific antibodies deliver a versatile palette of immunomodulatory and antitumor effects by uniting cellular cytotoxicity, immune activation, and targeted interference with pivotal signaling pathways, thereby establishing themselves as a flexible platform in modern immunotherapy. The realization of such a broad range of effects relies on interactions with specific populations of immune and tumor cells, which determine both the strength of the therapeutic response and the risk of adverse events.

### 2.3 Target cells for bispecific antibodies

The primary cellular targets of current bispecific antibodies (BsAbs) are T lymphocytes and natural killer (NK) cells. Their potent cytotoxicity and functional heterogeneity enable the induction of a multifaceted antitumor immune response.

T cells remain the central focus of BsAb-based therapies, particularly in the form of T-cell engagers that simultaneously bind CD3 and tumor-associated antigens. These constructs form artificial immunological synapses, activate MHC-independent signaling cascades, and induce the secretion of IFN-γ, TNF-α, perforin, and granzymes. BiTE molecules have demonstrated clinical efficacy in B-cell leukemia (29). Next-generation full-length bispecific antibodies—such as teclistamab, glofitamab, and tarlatamab—exhibit improved pharmacokinetics and have shown promising results in multiple myeloma, lymphomas, and lung cancer (1, 9). However, this approach carries risks including cytokine release syndrome (CRS), neurotoxicity, and reduced activity in “cold” tumor microenvironments. Current optimization strategies focus on two key aspects: first, the precise tuning of CD3-binding affinity to balance antitumor activity with minimal systemic toxicity; and second, the spatial engineering of the molecule to ensure optimal interdomain distance between antigen-binding sites, which is critical for effective synapse formation and selective T-cell activation within the tumor microenvironment.

NK cells are capable of MHC-independent killing of tumor cells. BsAbs targeting CD16A and tumor antigens initiate activation cascades involving SYK and PI3K and trigger the release of IFN-γ and TNF-α (44). AFM13 (CD30×CD16A) has shown efficacy in Hodgkin lymphoma (45), while AFM24 (EGFR×CD16A) is under investigation for the treatment of solid tumors (46). Trispecific molecules such as GTB-3550 (CD33×CD16A×IL-15) not only activate NK cells but also stimulate their proliferation (47). Critical parameters for development include tuning CD16A affinity to prevent NK-cell exhaustion and tailoring Fc domains depending on therapeutic goals. Despite advantages such as low risk of autoimmune complications, NK-cell–based approaches are limited by the short lifespan of effector cells and potential functional exhaustion.

γδ T cells are an MHC-independent T-cell subset activated via Vγ9Vδ2-TCR and NKG2D receptors (48). They recognize stress-induced ligands such as MICA and MICB and actively secrete proinflammatory cytokines. γδ T-cell engagers (GABs) that bridge γδ-TCRs and tumor antigens have demonstrated efficacy in preclinical models (49). However, in immunosuppressive microenvironments, γδ T cells may acquire regulatory properties and express inhibitory receptors such as PD-1 and LAG-3, limiting their antitumor function (50).

Macrophages and dendritic cells are gaining prominence as emerging targets in BsAb development. Tumor-associated macrophages (TAMs) frequently adopt an immunosuppressive M2 phenotype that promotes tumor progression. The CD47–SIRPα axis is a key therapeutic target; its blockade restores phagocytic activity (51, 52). BsAbs that co-target CD47 and tumor antigens (e.g., HER2) enable selective activation of macrophages while minimizing off-target effects on healthy cells (53). Alternative approaches include CSF1R inhibition, which promotes repolarization of TAMs toward an antitumor M1-like phenotype (54).

Dendritic cells, particularly the cDC1 subset, are essential initiators of adaptive immune responses. Targeting receptors such as CLEC9A and DEC-205 enables precise antigen delivery to cross-presentation compartments (55). Bispecific constructs incorporating CD40 specificity enhance DC maturation and promote IL-12 production, which is critical for the activation of Th1 cells and CD8^+^ cytotoxic T lymphocytes (56). These strategies offer a route to effective immune priming, even in tumors resistant to conventional therapies.

In summary, bispecific antibodies can be directed toward a wide range of immune cell types—from classical T and NK cells to less-characterized γδ T cells, macrophages, and dendritic cells. This versatility supports the design of multi-component therapeutic strategies that not only facilitate direct tumor eradication but also remodel the tumor immune microenvironment, thereby enhancing the overall efficacy of immunotherapeutic interventions. It is precisely the interplay between architecture, mechanisms of action, and cellular targets that determines the clinical efficacy of bispecific antibodies, as reflected in current examples of their application and in the prospects for further development.

### 2.4 Clinical applications and future perspectives of bispecific antibodies

Since their emergence in the late 20th century, bispecific T-cell engagers (BiTEs) have become a key therapeutic modality in hematologic malignancies. The original concept, proposed by Staerz and Bevan, demonstrated that hybrid antibodies capable of simultaneously binding CD3 and a tumor-associated antigen could induce apoptosis independently of MHC presentation (57). Blinatumomab (CD19×CD3) was the first FDA-approved agent in this class—initially in 2014 for minimal residual disease and later, in 2017, for relapsed or refractory acute lymphoblastic leukemia (ALL), achieving high rates of complete remission and improved survival outcomes (58, 59). Subsequently, full-length bispecific IgG molecules incorporating an Fc domain were developed to improve pharmacokinetics. Mosunetuzumab (CD20×CD3) was approved for follicular lymphoma after at least two prior lines of therapy, with overall response rates (ORR) reaching 80% (60). Teclistamab (BCMA×CD3) became the first bispecific antibody approved for relapsed/refractory multiple myeloma, with an ORR of approximately 63% (61). Similar agents include epcoritamab (CD20×CD3) and elranatamab (BCMA×CD3), both demonstrating significant activity in B-cell lymphomas and multiple myeloma (62, 63). Talquetamab (CD3×GPRC5D), the first therapeutic targeting GPRC5D, achieved an ORR of ~73% (64). Glofitamab (CD20×CD3), notable for its enhanced T-cell activation and finite treatment regimen (12 cycles), reported an ORR of 52% and complete remission rate (CR) of 39% in relapsed/refractory diffuse large B-cell lymphoma (DLBCL) (65).

In solid tumors, a milestone was the approval of tarlatamab (DLL3×CD3) for small-cell lung cancer. This agent, targeting DLL3, demonstrated an ORR of 40% and median overall survival of 14 months (66). Amivantamab (EGFR×MET), approved for non-small-cell lung cancer (NSCLC) with EGFR exon 20 insertions, combines dual receptor blockade with Fc-mediated cytotoxicity (39, 67).

Beyond oncology, BsAbs have therapeutic roles in other diseases. Emicizumab (FIXa×FX) was approved for prophylaxis in hemophilia A and represents the first subcutaneous, non-enzymatic agent capable of mimicking factor VIII activity (68).

To date, several bispecific antibodies have received FDA approval for clinical use, underscoring their therapeutic relevance and safety. These agents span both hematologic and solid malignancies, reflecting the diversity of targetable platforms. An overview of approved BsAbs is presented in **Table 1**.

**Table 1:** FDA-approved bispecific antibodies (as of July 2025).

Drug (trade name)	Targets	FDA-approved indications	Year approved	Disease type	Molecular format
Blinatumomab (Blincyto^®^)	CD19 × CD3	Acute lymphoblastic leukemia (MRD+, relapsed/refractory)	2014/2017	Hematologic malignancies	BiTE, Fc-free, intravenous administration
Teclistamab (Tecvayli™)	BCMA × CD3	Multiple myeloma (≥4 prior lines of therapy)	2022	Hematologic malignancies	IgG-like, DuoBody^®^
Mosunetuzumab (Lunsumio™)	CD20 × CD3	Follicular lymphoma (≥2 prior lines of therapy)	2022	Hematologic malignancies	IgG-like, DuoBody^®^
Epcoritamab (Epkinly™)	CD20 × CD3	Diffuse large B-cell lymphoma (relapsed/refractory)	2023	Hematologic malignancies	IgG1, subcutaneous administration
Elranatamab (Elrexfio™)	BCMA × CD3	Multiple myeloma (≥4 prior lines of therapy)	2023	Hematologic malignancies	IgG-like
Talquetamab (Talvey™)	GPRC5D × CD3	Multiple myeloma (≥4 prior lines of therapy)	2023	Hematologic malignancies	IgG-like
Glofitamab (Columvi^®^)	CD20 × CD3	Diffuse large B-cell lymphoma (fixed 12-cycle regimen)	2023	Hematologic malignancies	IgG-like, bivalent (2:1 CD20:CD3)
Tarlatamab (Imdelltra™)	DLL3 × CD3	Small cell lung cancer (relapsed/refractory)	2024	Solid tumors	IgG-like, T-cell engager
Amivantamab (Rybrevant^®^)	EGFR × MET	NSCLC (EGFR exon 20 insertion, post-chemotherapy)	2021	Solid tumors	IgG1, Fc-active, intravenous
Emicizumab (Hemlibra^®^)	FIXa × FX	Hemophilia A (with or without factor VIII inhibitors)	2017	Non-oncologic diseases	IgG-like, FVIIIa mimetic

• DLBCL, Diffuse large B-cell lymphoma

• ALL, Acute lymphoblastic leukemia

• BCMA, B-cell maturation antigen

• GPRC5D, Receptor overexpressed in myeloma

• DLL3, Notch ligand, specific to small cell lung cancer (SCLC)

• BiTE, Bispecific T-cell Engager (Fc-free format)

Development in bispecific immuno-oncology continues at a rapid pace. Zanidatamab (HER2×CD3) has demonstrated promising efficacy in HER2-positive gastrointestinal and breast cancers (69). Pasotuxizumab (PSMA×CD3) showed an ORR of up to 19% in castration-resistant prostate cancer (70), while zolbetuximab-CD3 (Claudin18.2×CD3) achieved an ORR of 28% in gastrointestinal malignancies (71). Zenocutuzumab (HER2×HER3) is showing encouraging activity in patients with NRG1 gene fusions (72). Advancements in multispecific platforms include the development of tri- and tetraspecific constructs with integrated cytokine modules to enhance antitumor responses. A recent example is the tetraspecific engager IPH6501, which combines CD20 targeting, NK cell activation (NKCE), and the delivery of a modified IL-2 variant. This construct demonstrates selective NK cell activation and robust antitumor activity in preclinical models of B-cell non-Hodgkin lymphomas, underscoring the promise of integrating cytokine signaling into multispecific platforms (73).

Numerous constructs are in late-stage clinical development, including CD20×CD3, BCMA×CD3, GPRC5D×CD3, and CD123×CD3, primarily for hematologic indications. In solid tumors, emerging agents are targeting HER2×HER3, PD-1/PD-L1, and CTLA-4. Several candidates exhibit favorable safety profiles (e.g., CRS ≤ grade 3) and convenient dosing schedules, including subcutaneous administration every 2–3 weeks (74).

In summary, bispecific antibodies have evolved from experimental prototypes into an established therapeutic class with broad clinical applicability. By enabling targeted cytotoxicity independent of MHC expression, they offer promising new avenues for the treatment of tumors resistant to conventional therapies. The evolution of antibody formats has laid the foundation for the emergence of new directions in bispecific immunotherapy that extend beyond classical architectures, thereby overcoming the limitations of conventional antibodies, broadening the range of therapeutic targets, and creating the prerequisites for the development of more universal and adaptive immunotherapeutic strategies.

## 3 Bispecific T-cell receptor-based constructs

### 3.1 Design principles and mechanisms of action

Bispecific antibodies based on T-cell receptors (TCR-like and TCR-engineered) represent an innovative class of molecules capable of recognizing intracellular tumor antigens presented in the context of peptide–MHC complexes. These constructs overcome the limitations of conventional antibodies, which are restricted to targeting surface antigens, by mimicking the specificity of native TCRs. Their binding domains are typically composed of scFv or Fab fragments that have been engineered to enhance affinity and specificity (75, 76). The development of such molecules relies on phage display, directed evolution, and CDR optimization, with careful attention to cross-reactivity, which remains a critical concern (77, 78).

Their mechanism of action involves selective binding to tumor-specific peptide–MHC complexes, leading to the formation of an immune synapse and subsequent T-cell activation. Engagement of intracellular signaling cascades (ZAP70, LAT, PLCγ1) culminates in the expression of transcription factors such as NFAT and secretion of cytotoxic effectors (79–81). This approach is particularly effective for targeting tumor-specific peptides—such as WT1, MAGE-A3, and NY-ESO-1—when presented in the context of HLA-A*02:01 (82–84).

TCR-like constructs require rigorous validation to confirm allele specificity and ensure safety. Variations in peptide sequence or MHC allele can significantly affect binding, necessitating the use of immunopeptidomics and normal tissue screening to identify potential off-target interactions (85–87). While enhanced affinity facilitates the detection of low-abundance targets, excessive affinity may increase the risk of off-tumor toxicity (88, 89).

Stability and pharmacologic performance can be improved through site-directed mutagenesis and rational design, including the replacement of hydrophobic residues, modification of CDR loops, and the development of prodrug formats that are selectively activated in the tumor microenvironment (90–92). The applicability of TCR-like antibodies is constrained by their reliance on specific HLA alleles, which has prompted the development of broadly applicable constructs focused on frequent alleles—especially HLA-A*02:01 (93, 94).

The most advanced clinical platform is ImmTAC, which employs engineered TCRs fused to an anti-CD3 effector domain for T-cell engagement (95, 96). ImmTAC molecules are characterized by high sensitivity to peptide–MHC complexes and have demonstrated activity against targets with low expression. A landmark example is tebentafusp, which received FDA approval for metastatic uveal melanoma and significantly improved overall survival in a Phase III clinical trial (97–99).

In conclusion, TCR-based bispecific antibodies offer a unique therapeutic modality by targeting intracellular tumor antigens restricted by defined MHC alleles. The application of advanced protein engineering strategies to enhance affinity, stability, selectivity, and pharmacokinetics enables the generation of potent and safe therapeutic agents. In the context of personalized oncology, these constructs hold great promise as integral components of next-generation combination immunotherapies.

### 3.2 Preclinical and clinical examples

The development of TCR-like and TCR-engineered bispecific antibodies has opened new avenues for cancer immunotherapy by enabling the recognition of intracellular antigens presented in complex with MHC molecules. These constructs significantly broaden the therapeutic landscape compared to conventional BsAbs, which are limited to surface antigens. One of the earliest examples was ESK1, a TCR-like antibody specific for WT1 in the context of HLA-A*02:01. In preclinical models, ESK1 demonstrated selective cytotoxicity and a favorable safety profile, while clinical studies confirmed its capacity for specific tumor targeting *in vivo* (82, 100).

The most clinically advanced and successful example to date is tebentafusp, an ImmTAC molecule directed against gp100/HLA-A*02:01. By combining an affinity-enhanced TCR domain with an anti-CD3 effector arm, tebentafusp enables potent T-cell recruitment and tumor control. In a pivotal Phase III trial, tebentafusp significantly improved overall survival in patients with metastatic uveal melanoma, marking the first effective therapy for this disease (97–99).

High efficacy has also been demonstrated in preclinical systems for TCR-based bispecific antibodies targeting cancer-testis antigens such as NY-ESO-1 and MAGE-A1. These antigens, which exhibit restricted expression in normal tissues, were shown to be immunogenic and safe targets; specific constructs elicited robust T-cell responses with minimal toxicity (101–103). Similar results were obtained with molecules targeting PRAME—an oncogenic antigen broadly expressed in solid tumors. PRAME/HLA-A*02:01-specific constructs demonstrated selective cytotoxicity and promising clinical activity (104, 105).

Targeting MART-1 has served as a benchmark for demonstrating fine-tuned TCR engineering. Mutagenesis of CDR loops and optimization of framework regions significantly enhanced binding selectivity for tumor-associated epitopes while minimizing recognition of normal melanocytes (106, 107). Comparable engineering strategies are being employed to develop constructs against AFP, mutant p53, and viral epitopes such as HPV E6, enabling applications in virus-associated cancers (75, 108, 109).

The accumulated preclinical and clinical data strongly support the potential of TCR-based bispecific platforms as a next-generation modality in personalized immunotherapy. By accessing previously undruggable intracellular targets, these constructs offer the opportunity to significantly expand the therapeutic arsenal in oncology. At the same time, progress in the field of TCR-bispecific constructs is accompanied by the need to address practical challenges related to their production, stability, and reproducibility, which directly determine the successful implementation of these molecules in clinical practice.

### 3.3 Manufacturing and stability challenges of TCR-based bispecific constructs

The development of bispecific antibodies based on T-cell receptors (TCR-like and TCR-engineered) is accompanied by a set of unique bioengineering and manufacturing challenges. A primary hurdle is achieving high specificity for peptide–MHC complexes while minimizing cross-reactivity. This necessitates multi-step optimization workflows that often employ phage or yeast display platforms for candidate selection. Nevertheless, even well-characterized molecules require extensive preclinical validation using panels of normal tissues and immunopeptidome libraries to assess potential off-target interactions (95, 110).

Another major challenge lies in the intrinsic instability of TCR domains, which are prone to aggregation and misfolding. To mitigate these issues, structural engineering is employed, including the introduction of stabilizing mutations and disulfide bridges, as well as careful selection of molecular formats—such as scFv, Fab, or IgG scaffolds—that support native folding and thermal stability (111–113). Aggregation remains a particularly critical issue, as it reduces bioavailability, increases immunogenicity, and complicates biomanufacturing. Solutions include rational mutagenesis and optimization of buffer composition for long-term storage (114, 115).

Immunogenicity represents an additional obstacle. Modified TCR domains may elicit anti-drug antibody (ADA) responses, thereby reducing therapeutic efficacy. The use of humanized sequences and minimization of non-human epitopes are key strategies to reduce immunogenic potential (90, 113, 116).

At the manufacturing scale-up stage, particular attention must be given to molecular stability at high protein concentrations required for therapeutic dosing. TCR-based platforms are prone to aggregation at concentrations above 50 mg/mL due to surface hydrophobicity. This necessitates customized buffer systems, stabilizing excipients, and optimized concentration protocols (117, 118).

For transportation and storage, resistance to physicochemical and mechanical stress is essential. Strategies such as formulation with arginine hydrochloride and amino acid-based stabilizers, the use of silicone-free syringes, and lyophilization techniques help maintain molecular integrity during long-term storage (109).

In summary, the successful clinical implementation of TCR-like and TCR-engineered bispecific antibodies requires the integration of structural biology, pharmaceutical formulation science, and rigorous quality control at every stage. Overcoming challenges related to stability, immunogenicity, and manufacturing will be essential for the widespread adoption of these novel immunotherapeutic agents. Alongside manufacturing and pharmaceutical aspects, the clinical specificity of applying TCR-like and TCR-engineered constructs is of fundamental importance, as it markedly distinguishes them from classical bispecific antibodies and defines both the opportunities and the limitations of this approach.

### 3.4 Clinical differences between TCR-like/TCR-engineered and conventional bispecific antibodies

Clinical distinctions between TCR-like or TCR-engineered bispecific antibodies and conventional BsAbs become particularly evident when comparing their mechanisms of action, efficacy profiles, and safety characteristics. Traditional BsAbs, such as blinatumomab, exhibit strong activity against tumors expressing high-density surface antigens—an attribute exemplified by their success in B-cell acute lymphoblastic leukemia (B-ALL) (28, 57, 58). However, their therapeutic effect is highly dependent on the stability and density of antigen expression, rendering them less effective in the context of antigen loss or downregulation. In contrast, TCR-like and TCR-engineered antibodies are capable of recognizing intracellular tumor-derived peptides presented by MHC molecules. This enables targeting of non-surface antigens, including neoantigens and viral proteins, expanding their applicability to tumors with low surface antigen expression or concealed immunogenic profiles—such as sarcomas, melanoma, and select hematologic malignancies (109, 119). A prime example is tebentafusp, which has demonstrated a statistically significant survival benefit in patients with metastatic uveal melanoma (97, 99). IMA203, a TCR-engineered T-cell therapy targeting PRAME in HLA-A*02^+^ patients with relapsed or refractory solid tumors, demonstrated a favorable safety profile in a phase I study (NCT03686124), with no dose-limiting toxicity, a low incidence of severe CRS, and absence of neurotoxicity. Among 41 enrolled patients, the overall response rate was 52.5% and the confirmed objective response rate was 28.9%, indicating the promise of this approach for the treatment of melanoma and sarcomas (120).

The safety profiles of these platforms also differ markedly. Conventional BsAbs—especially BiTEs—are associated with high rates of cytokine release syndrome (CRS) and neurotoxicity due to broad polyclonal T-cell activation. For instance, CRS has been observed in up to 70% of blinatumomab-treated patients, with neurotoxic events occurring in 15–20% of cases (121). In contrast, TCR-like constructs typically do not induce systemic T-cell activation but carry distinct risks related to cross-reactivity with peptides derived from normal tissues. Severe toxicities, including myositis and cardiotoxicity, have been reported for TCR domains targeting MAGE-A3 (77, 122).

Modern engineering approaches help mitigate these risks through fine-tuning of affinity, optimization of CDR regions, and multilayered specificity validation. Constructs targeting WT1, PRAME, and NY-ESO-1 have demonstrated that careful molecular design can achieve high selectivity with acceptable safety profiles (82, 103, 123).

Despite their technical complexity, cost of production, and the requirement for HLA-matched patient populations, TCR-like agents represent a critical addition to the immunotherapy arsenal.

The future of both BsAb strategies lies in the development of multispecific formats, improved antigen selectivity, and the refinement of predictive markers for toxicity and immunogenicity. Together, conventional and TCR-based bispecific antibodies form a complementary toolkit for precision immuno-oncology, adaptable to the molecular profile of individual tumors and tailored to patient-specific therapeutic needs.

## 4 Bispecific aptamers: design principles and therapeutic potential

### 4.1 Structure and advantages of aptamers

Aptamers are short, single-stranded DNA or RNA molecules capable of folding into stable three-dimensional structures—such as hairpins, pseudoknots, and G-quadruplexes—that enable high-affinity and highly specific binding to molecular targets, including proteins, peptides, nucleic acids, and small molecules. Owing to their compact architecture and structural flexibility, aptamers are emerging as attractive alternatives to antibodies for applications in targeted delivery and molecular recognition (124–126).

A key advantage of aptamers lies in their selection method—SELEX (Systematic Evolution of Ligands by EXponential enrichment)—which allows for the *in vitro* identification of high-affinity binders from vast nucleotide libraries. Unlike antibodies, aptamers do not require expression in living cells, simplifying production, improving purity, minimizing batch-to-batch variability, and facilitating scalability at lower cost (127). With low molecular weights (5–15 kDa), aptamers exhibit rapid tissue penetration—including into tumors—and are well-suited for diagnostic and therapeutic applications. However, their small size also leads to rapid renal clearance and short circulation half-lives. Strategies such as PEGylation, albumin-binding, and nanoparticle encapsulation are employed to extend systemic persistence (128). Additionally, chemical modifications—such as 2′-fluoro substitutions or phosphorothioate linkages—enhance nuclease resistance and reduce immunogenicity (129, 130).

Although aptamers are not traditionally bispecific agents, they can be engineered to perform bispecific functions. Bispecific aptamers (bsApts) are synthetic oligonucleotides designed to bind two distinct targets simultaneously—typically a tumor-associated antigen and an immune-cell receptor (e.g., CD3, CD28, 4-1BB). They represent a promising alternative to antibodies, particularly where small size and low immunogenicity are advantageous. Recent advances in molecular engineering have enabled the conversion of monospecific aptamers into bispecific constructs, thereby expanding their utility in immunotherapy.

Key engineering strategies include bivalent aptamer formats and hybrid aptamer–antibody fusions. For example, bivalent bsApts targeting immune checkpoints such as PD-1 and CTLA-4 have demonstrated synergistic effects in reactivating T-cell responses. These constructs retain core aptamer advantages—small size and low immunogenicity—while acquiring bispecific antibody-like functionality (131, 132). In hybrid designs, aptamer modules specific to tumor antigens are fused to antibody fragments (e.g., anti-CD3 scFv), enabling the recruitment of T cells in a manner analogous to BiTEs. Preclinical models have validated their tumor-directed cytotoxic potential (133).

The valency of bsApts is often denoted as [m + n], where [m] is the number of tumor-targeting modules and [n] denotes immune-cell engagement domains. For example, [1 + 1] constructs (e.g., c-Met/CD16A) are monovalent on both ends, while [1 + 2] (PSMA/4-1BB) and [2 + 2] (MUC1/CD16A) designs exhibit enhanced avidity. Linker length is critical: optimal spacers range from 7 to 22 nucleotides (~49–152 Å), aligning with physiological immune synapse dimensions. Longer linkers (>29 nucleotides) reduce efficacy, and linker rigidity matters—double-stranded segments provide synaptic stability superior to flexible single-stranded linkers (134).

Compared with antibodies, bispecific aptamers (bsApts) offer several advantages: extremely low immunogenicity, no Fc-mediated effects (such as ADCC or cytokine-release syndrome), cell-free chemical synthesis, and straightforward chemical modification. Each of the three platforms—classical bispecific antibodies, TCR-based bispecific constructs, and bispecific aptamers—has its own strengths and limitations. Antibodies provide well-characterized pharmacokinetics and standardized manufacturing, yet they are prone to CRS and limited tissue penetration. TCR-based constructs grant access to intracellular neoantigens but remain HLA-restricted and more immunogenic. Aptamers exhibit excellent tissue diffusion and chemical flexibility, although they have not yet achieved clinical validation. A side-by-side comparison of all three platforms is presented in **Table 2**.

**Table 2:** Comparison of Therapeutic Bispecific Platforms.

Criterion	Classical bispecific antibodies	TCR-like/TCR-engineered bispecific antibodies	Bispecific aptamers
Typical targets	Surface antigens (CD19, HER2, BCMA)	Peptide–MHC complexes (WT1, PRAME, MAGE-A3)	Surface/soluble antigens (PSMA, c-Met) + immune receptors (CD3, CD16, 4-1BB)
MHC dependency	No	Yes	No
Antigen type	Extracellular	Intracellular peptides	Extracellular (SELEX-adaptable)
Molecular size	≈150 kDa (IgG)/≈55 kDa (BiTE)	55–110 kDa (format-dependent)	5–40 kDa
Clinical status	Several FDA approvals; hematologic and some solid tumors	One FDA approval (tebentafusp); HLA-restricted trials	Preclinical/early clinical; no approvals
Immunogenicity	Moderate	Variable	Very low
Pharmacokinetics	Half-life days–weeks (Fc-bearing)	Hours–days; less stable	Minutes–hours; extendable via PEG/albumin
Tissue penetration	Moderate; limited by stroma	Moderate; stroma-limited	High due to small size
Manufacturing	Established CHO/HEK platforms	Complex engineering, aggregation issues	Solid-phase chemical synthesis
Manufacturing Cost	High	Extremely high	Low
Key toxicity risks	CRS, neurotoxicity, on-target/off-tumor effects	Cross-reactivity, organ toxicity	Minimal Fc-associated effects; potential off-target binding

Despite their substantial preclinical promise, clinical translation of bsApts remains limited, mainly due to the need for more comprehensive studies on their pharmacokinetics, biodistribution, and long-term safety. Nonetheless, their potential as a flexible, non-immunogenic platform for bispecific agent development positions them as a compelling alternative to traditional antibody therapies. These unique properties of aptamers are reflected in the specific mechanisms of action of bispecific constructs, which define their therapeutic potential and distinguish them from antibody-based platforms.

### 4.2 Mechanisms of action of bispecific aptamer constructs

Bispecific aptamers (bsApts) exert antitumor activity primarily through the formation of artificial immune synapses between tumor and immune cells (135, 136). Upon simultaneous binding to a tumor-associated antigen (e.g., PSMA, c-Met, or MUC1) and a receptor on an immune effector cell (e.g., CD3, CD16, or 4-1BB), bsApt molecules bring the cells into close proximity, mimicking a natural immune synapse (137, 138). This induces cytotoxic responses through perforin and granzyme release, and the activation of apoptosis via FasL/Fas and TNFα/TNFR signaling pathways.

BsApts bypass the need for MHC–peptide interaction, which is particularly advantageous in tumors with low or absent MHC class I expression (139). Aptamers targeting co-stimulatory receptors such as CD28 (140), 4-1BB (141), or OX40 (142) further enhance T-cell activation by providing secondary activation signals. Constructs like c-Met/CD16a can also engage NK cells and γδ T lymphocytes, triggering ADCC (135, 143). The efficacy of these constructs depends on spatial parameters: linkers of 7–22 nucleotides (~150 Å) allow for optimal intercellular distances and synapse formation (135). Multivalent formats such as [2 + 2] MUC1/CD16a exhibit enhanced avidity and interaction stability (144).

A separate class of bsApts functions as immune checkpoint antagonists. Constructs targeting PD-1 (145, 146), CTLA-4 (147), and TIM-3 (148) have shown the ability to reactivate exhausted T cells. Combining bsApts with complementary mechanisms—such as co-stimulation and checkpoint blockade—can yield synergistic effects (149). Another promising direction involves the design of multispecific constructs that target multiple tumor antigens simultaneously (150, 151), potentially improving selectivity and therapeutic efficacy.

Additionally, bsApt platforms can serve in targeted drug delivery, where one domain ensures selective cell binding and the other captures and transports therapeutic agents or nanoparticles (125). The mechanisms discussed find both confirmation and practical implementation in a number of preclinical and clinical studies, demonstrating the potential of bispecific aptamers as next-generation therapeutic agents.

### 4.3 Preclinical and clinical development of bispecific aptamers

Over recent years, several bispecific aptamer constructs have demonstrated notable efficacy in preclinical studies. One of the earliest and most studied examples is the c-Met/CD16a construct, composed of DNA aptamers targeting c-Met on tumor cells and FcγRIII (CD16a) on NK cells. *In vitro*, this bsApt induced ADCC-mediated lysis of gastric and lung cancer cells, comparable to cetuximab (135). Optimization of the linker length (7–22 nucleotides) proved essential for effective synapse formation.

Another important example is the PSMA/4-1BB bsApt, which combines a 2′-fluoro RNA aptamer against PSMA with a dimeric aptamer targeting 4-1BB. In colorectal and melanoma models, it suppressed tumor growth and metastasis at doses tenfold lower than corresponding monospecific agents (140). A construct targeting MRP1/CD28 was developed to activate T cells against chemoresistant melanoma stem-like cells and enhanced the efficacy of GVAX vaccination when combined with Foxp3 suppression (152). A tetravalent MUC1/CD16a bsApt showed high avidity and selectively lysed MUC1-positive A549 cells, sparing MUC1-negative HepG2 cells (144).

Among the clinically advanced aptamers, most target cytokines. These include NOX-E36 (anti-CCL2) and NOX-A12 (anti-CXCL12), both explored in immunomodulatory contexts (153). A novel bsApt, Ap3-7c, was developed to simultaneously block PD-1/PD-L1 interactions and facilitate physical contact between T cells and tumor cells. Ap3-7c employs a “recognition-then-conjugation” mechanism in which the aptamer covalently anchors to its target, prolonging residence time in the tumor microenvironment and enhancing therapeutic efficacy (9).

AYA227 is a newly developed bifunctional aptamer targeting both CTLA-4 and NKG2A. Designed with machine learning algorithms, it activates both T and NK cells and demonstrates the potential to overcome immune suppression in solid tumors (154).

Another approach involves a bsApt targeting pancreatic tumors, conjugated with the cytotoxic agent monomethyl auristatin E (MMAE). The addition of a universal antibody fragment for delivery extended the *in vivo* half-life and reduced systemic toxicity (155).

In clinical settings, the aptamer AS1411, targeting nucleolin, completed a Phase II trial for metastatic renal cell carcinoma (156). Although it demonstrated safety and moderate efficacy, its therapeutic window was limited by insufficient specificity in heterogeneous tumors. Ongoing studies, such as the Phase I/II GLORIA trial, are exploring the combination of aptamers with radiotherapy for glioblastoma, highlighting the potential of bsApts in multimodal regimens (157).

Despite significant progress, key challenges remain—particularly in optimizing *in vivo* stability and overcoming antigenic heterogeneity. Nevertheless, the modularity and adaptability of bispecific aptamers offer strong potential for the development of personalized therapeutic strategies, especially when integrated with artificial intelligence approaches for structure prediction and optimization. The accumulated preclinical and clinical data not only confirm the therapeutic validity of bispecific aptamers but also reveal a number of limitations, the understanding of which is key to defining the future prospects of this platform.

### 4.4 Limitations and future prospects of bispecific aptamers

Despite the considerable potential of bispecific aptamers (bsApts) in cancer therapy, several major limitations currently hinder their clinical translation. The foremost challenge is their short *in vivo* half-life, primarily due to rapid degradation by nucleases and renal clearance associated with their small size and polyanionic nature (125, 158, 159). This necessitates frequent dosing, which reduces therapeutic convenience and increases patient burden. While bsApts exhibit lower immunogenicity compared to antibodies (160), their long-term immunological impact remains insufficiently characterized.

Another critical challenge is achieving an optimal balance between binding specificity and affinity (161, 162). In highly heterogeneous tumor microenvironments, multivalent aptamers may exhibit undesired cross-reactivity, leading to off-target effects (163). For targets such as PSMA, rapid internalization after ligand binding complicates the formation of a stable immune synapse (164). Furthermore, large multivalent constructs often face poor tissue penetration, particularly in dense solid tumors (165). The inherent susceptibility of nucleic acids to nuclease degradation poses additional obstacles, particularly in indications such as glioblastoma, where traversal of the blood–brain barrier is required (166, 167). Moreover, the complexity of large-scale manufacturing and quality control of multivalent aptamer constructs continues to limit their clinical development.

Nonetheless, bsApts possess unique advantages—including ease of chemical modification, low immunogenicity, and precise tumor targeting—that underpin their future therapeutic potential. Several promising directions are currently being pursued. One is the development of conditionally activated constructs responsive to tumor microenvironment cues such as pH, redox status, or protease activity, which could enhance tumor specificity (168). Aptamers targeting emerging immune checkpoints (e.g., TIGIT, VISTA, B7-H3, CD73) are being explored to overcome resistance to current immunotherapies (169). Multispecific aptamers capable of binding multiple tumor antigens (e.g., EpCAM and CD44) offer increased specificity and reduced toxicity (170).

Combining bsApts with other therapeutic modalities—such as photosensitizers, chemotherapeutics, or gene-editing tools—opens avenues for multimodal platforms (171). Pharmacokinetic properties may be improved through nucleotide modifications (e.g., 2′-fluoro, 2′-O-methyl, PEGylation) (159) and the incorporation of delivery systems including liposomes, exosomes, and metal–organic frameworks (172).

Particularly promising is the integration of artificial intelligence for aptamer structure prediction and design optimization (173), alongside multi-omics approaches for precise target identification. The development of universal platforms for bsApt construction (155) and their potential combination with CAR T cells (174) could accelerate the adoption of personalized therapeutic regimens. Furthermore, the use of bsApts for targeted delivery of therapeutic agents—such as siRNAs or cytotoxic drugs—offers additional opportunities for cancer treatment (150).

Realizing the full therapeutic potential of bsApts will require multidisciplinary collaboration across structural biology, medicinal chemistry, pharmacology, and clinical oncology to overcome current barriers and translate these promising molecules into effective, patient-specific therapies.

Thus, the development of bispecific formats—from classical antibody constructs to TCR molecules and aptamer-based platforms—demonstrates significant progress in expanding the arsenal of immunotherapeutic agents. Each of these approaches possesses its own advantages and limitations. However, the broadening of the immunotherapy toolkit is inevitably accompanied by new challenges, including adverse effects, toxicities, and manufacturing constraints, which become key considerations in assessing the true clinical potential of bispecific agents.

## 5 Adverse effects and limitations of bispecific therapy

### 5.1 Off-tumor toxicity

Bispecific antibodies (BsAbs), TCR-based constructs, and bispecific aptamers represent promising classes of immunotherapeutic agents designed to enhance antitumor immune responses (2). The dual specificity underlying these constructs allows for the simultaneous recognition of a tumor antigen and an activating receptor on an immune effector cell. However, when the target antigen is also expressed—albeit at low levels—on normal tissues, this can result in “on-target/off-tumor” toxicity. Such effects may include cytokine release syndrome (CRS), organ damage, neurologic complications, and other serious adverse reactions that not only reduce therapeutic efficacy but may also pose life-threatening risks.

BsAbs, particularly bispecific T-cell engagers (BiTEs), function by linking tumor antigens with CD3 on T lymphocytes, triggering immune activation (28, 175). While these molecules have shown remarkable efficacy in hematologic malignancies, they are also associated with significant off-tumor toxicity. For example, blinatumomab—a CD19×CD3 BsAb—induces CRS in the majority of patients with acute lymphoblastic leukemia (ALL). Clinical studies report that 90% of patients experience fever, 60% develop hypotension, and 20% present with severe respiratory distress requiring intensive care (57). Moreover, neurological adverse events—including encephalopathy, seizures, and speech disturbances—occur in over 50% of treated patients and are thought to result from CD19 expression on certain neuronal subpopulations (174, 176).

In the context of solid tumors, CD3-directed BsAbs targeting antigens such as CEA, EpCAM, and HLA-A*02:01:gp100 have produced dose-dependent toxicity (177–180). Observed toxicities included hepatotoxicity, intestinal inflammation, systemic cytokine responses, gastrointestinal and respiratory dysfunctions, and cardiovascular abnormalities. Although some of these effects may be linked to localized tumor inflammation, most were reversible upon treatment discontinuation. One effective mitigation strategy has been reducing the affinity of the CD3-binding domain to limit activation in normal tissues.

A notable example of successful toxicity optimization is amivantamab (EGFR×MET), a BsAb designed to simultaneously target two tumor-specific receptors. This dual-targeting approach reduced off-tumor side effects by over 40% without compromising antitumor activity (181). The rationale lies in the fact that co-expression of both antigens is more common in malignant cells than in healthy tissues, enhancing selectivity.

Spatial configuration of antigen-binding domains also plays a role in minimizing toxicity. Rational positioning of Fab fragments within the BsAb structure has been shown to significantly reduce cross-reactivity with normal tissues by improving selective cell engagement (182).

A particularly innovative approach involves the development of “masked” BsAbs, which remain inactive in circulation and are only activated by tumor-specific proteases within the tumor microenvironment. This platform, described in detail in (183, 184), offers dual protection: first, by preventing interaction with healthy tissue, and second, by ensuring localized activation in neoplastic zones where protease concentrations are elevated.

CRS mitigation strategies include step-up dosing regimens and subcutaneous administration, as demonstrated in trials of glofitamab and teclistamab (both CD20×CD3). These approaches significantly reduced the incidence of severe CRS from 45% to less than 15% (185, 186), enabling more controlled immune activation and avoiding massive cytokine surges.

TCR-based soluble constructs, unlike BsAbs, recognize peptides presented by HLA molecules rather than surface antigens. While this expands the repertoire of intracellular tumor targets, it also increases the risk of cross-reactivity with normal tissues. A critical example is a clinical trial targeting MAGE-A3, where 33% of patients developed fatal myocarditis due to cross-recognition of titin in cardiac muscle (76). Similarly, a TCR targeting CEA led to severe colitis in 70% of colorectal cancer patients due to antigen expression in intestinal epithelium (187).

Comparative analysis of toxicity profiles reveals fundamental differences between BsAbs and TCR-based molecules. BsAbs are primarily associated with systemic effects (e.g., CRS, hematologic toxicity), whereas TCR constructs more commonly cause organ-specific damage aligned with tissue antigen expression. Current strategies to reduce TCR-related toxicity include in silico modeling of cross-reactivity (188, 189) and selection of antigens with highly restricted expression in healthy tissues (190, 191).

Immune effector cell-associated neurotoxicity syndrome (ICANS), although less well understood, is a serious complication linked to blood–brain barrier (BBB) disruption. Experimental evidence indicates that TCR-mediated activation of cerebral microvascular endothelium increases BBB permeability, facilitating infiltration of both cytokines (e.g., IL-1β, IFN-γ) and activated T cells into the CNS (192). Clinicopathologic studies confirm the presence of perivascular lymphocytic infiltration in patients with severe neurologic symptoms (193).

In summary, while bispecific constructs hold significant therapeutic promise, off-tumor toxicity remains a major limitation to their clinical use. Addressing this challenge will require a multifaceted approach encompassing rational molecule design, dose optimization, targeted activation strategies, and robust management protocols for adverse events.

### 5.2 Physicochemical barriers to tumor penetration

The therapeutic efficacy of bispecific constructs in solid tumors is substantially limited by a series of fundamental physicochemical barriers that impair their intratumoral penetration. The first and most critical obstacle is the abnormal tumor vasculature, which is characterized by disorganized architecture, elevated permeability, and irregular blood flow. Dynamic contrast-enhanced MRI (DCE-MRI) studies have shown that only 0.001–0.01% of the administered antibody dose reaches tumor tissue, a phenomenon referred to as the “perfusion effect” (194). This limitation is particularly pronounced for large molecules, such as IgG-like bispecific antibodies (~150 kDa), which demonstrate significantly poorer tissue penetration compared to small-molecule agents (195). The transport of bispecific constructs to tumor niches is influenced not only by molecular weight but also by conformational flexibility. Neutron scattering experiments have shown that tetravalent IgG/scFv formats exhibit an effective hydrodynamic radius of 8–10 nm, whereas more compact diabody constructs achieve 4.5 nm, resulting in a twofold increase in diffusion coefficients through type I collagen matrices (196).

A second major limitation is elevated interstitial fluid pressure (IFP), which can reach 40–60 mmHg in the core of large tumors, creating a pressure gradient that impedes the diffusion of macromolecules (197). The dense extracellular matrix (ECM)—rich in type I collagen, hyaluronic acid, and fibronectin—acts as an additional diffusion barrier, restricting the intratumoral spread of bispecific agents. Immunohistochemical analysis of biopsy samples from patients treated with bispecific antibodies has revealed strong perivascular accumulation with minimal penetration into the tumor parenchyma (198). Blockade of VEGF using anti-VEGF or anti-VEGFR2 antibodies has been shown to enhance both BsAb infiltration and CD8^+^ TIL accumulation in preclinical models, thereby increasing antitumor efficacy (199). This challenge is particularly severe in desmoplastic tumors such as pancreatic ductal adenocarcinoma, where ECM density can reduce antibody diffusion by an order of magnitude compared to less fibrotic tumors.

Penetration through the blood–brain barrier (BBB) represents another major challenge in treating CNS metastases. Pharmacokinetic studies show that bispecific antibody concentrations in cerebrospinal fluid are typically <0.1% of plasma levels, severely limiting therapeutic impact in brain lesions (200). Preclinical evaluation of the anti-EGFRvIII BsAb AMG 596 in glioblastoma models confirmed minimal brain tumor penetration, prompting the development of BBB-crossing strategies (201).

To address these barriers, several strategies have been developed to improve tumor penetration. One approach involves reducing molecular size via monovalent or fragmented constructs. Studies using F(ab’)_2_ fragments of bispecific antibodies have shown 5–10-fold increases in tissue persistence (202). Another promising method involves enzymatic modulation of the ECM using agents such as hyaluronidase or collagenase. In clinical trials, PEGPH20 (a pegylated hyaluronidase) combined with anti–PD-L1 antibodies significantly enhanced intratumoral distribution of immunotherapeutic agents (203).

Protease-activatable BsAbs represent an additional innovative solution, remaining inert in circulation and becoming activated only within the protease-rich tumor microenvironment (204).

In summary, optimizing the design and delivery of bispecific constructs—through molecular size reduction, tumor microenvironment modulation, and conditional activation strategies—offers a path to partially overcome these physicochemical barriers. However, achieving clinically meaningful improvements in penetration, particularly in fibrotic or immune-privileged tumor niches, will require further refinement of molecular architecture informed by tumor-specific biology.

### 5.3 Antigenic drift

Bispecific antibodies (BsAbs), TCR-like constructs, and aptamer-based platforms offer significant therapeutic promise in cancer immunotherapy. However, their clinical efficacy is often undermined by antigenic drift—a dynamic process wherein the expression or structure of the target antigen evolves under therapeutic selective pressure. Analogous to mechanisms observed in infectious diseases, antigenic drift enables tumor cells to escape immune surveillance and develop resistance. Key mechanisms include epigenetic silencing, genomic deletions, alternative splicing, and post-translational modifications (205), all of which reduce the effectiveness of monospecific or narrowly targeted bispecific agents.

Positron emission tomography (PET) studies using ^89Zr-labeled trastuzumab revealed that, even in HER2-overexpressing breast tumors, antibody distribution is markedly heterogeneous, with the formation of “pharmacological sanctuaries” nearly devoid of drug accumulation (206, 207). In hematologic malignancies, up to 30–50% of patients treated with blinatumomab (CD19×CD3) relapse due to CD19 antigen loss, as confirmed by immunohistochemistry and flow cytometry (208). A similar phenomenon has been observed with AMG 330 (CD33×CD3) in acute myeloid leukemia, where CD33-low/negative clones were detected in ~60% of patients who initially responded but later relapsed (209).

In solid tumors, spatial heterogeneity of antigen expression further exacerbates this issue. Analyses of biopsies before and after treatment with BsAbs targeting EGFR (amivantamab) or HER2 (zintokalimab) have demonstrated substantial intercellular variation in antigen density within single tumor niches (210–212).

Antigenic drift poses a particular challenge when targeting neoantigens such as EGFRvIII in glioblastoma or KRAS G12D in pancreatic adenocarcinoma. Clinical studies of the EGFRvIII-targeting BsAb AMG 596 showed that within 12 weeks of treatment, 70% of patients had downregulated the target epitope via selective expansion of EGFRvIII-negative subclones (201). Similar immune escape has been reported with TCR-like constructs directed against mutant p53 peptides, where loss of HLA alleles or defects in antigen processing machinery (e.g., TAP, β2-microglobulin) allowed tumor evasion (213).

Several strategies have been proposed to counteract antigenic drift. One involves dual targeting of tumor antigens—such as in amivantamab (EGFR×MET)—which reduced antigen-loss–associated relapse from 45% to 15% compared to monospecific agents (214). An alternative is targeting constitutively expressed tumor-maintenance antigens, such as B7-H3 or Claudin 6, which are less prone to downregulation (215). Targeting components of the tumor stroma (e.g., FAP, PDGFRβ), which exhibit lower antigenic variability, is also under investigation (216). Still, none of these approaches fully eliminate the risk of immune escape, highlighting the need for real-time monitoring of the tumor antigenic landscape and adaptive therapeutic adjustment.

### 5.4 Immunogenicity and anti-drug antibodies

The induction of anti-drug antibodies (ADAs) represents a significant limitation for the clinical application of bispecific constructs, adversely affecting pharmacokinetics, efficacy, and safety. In addition to neutralizing therapeutic activity, ADA formation can lead to hypersensitivity reactions and immune complex–mediated toxicity (217, 218).

Immunogenicity arises from the presence of non-self elements, such as murine or chimeric antibody sequences, artificial peptide linkers, and non-natural spatial arrangements of antigen-binding domains. ADA incidence varies by platform: 5–15% for full-length IgG-like BsAbs and up to 30–60% for small formats like BiTEs and DARTs (2).

Due to the relatively recent clinical adoption of BsAbs, long-term immunogenicity data are limited. For blinatumomab, ADA formation occurs in less than 1% of patients (219). Similar findings apply to BsAbs targeting B-cell markers—such as mosunetuzumab (IgG 1 + 1, CD20×CD3) and glofitamab (IgG 2 + 1, CD20×CD3)—where ADA development has not been observed regardless of format (217). This may be attributed to B-cell depletion, which prevents the generation of a humoral immune response against the therapeutic antibody.

Indeed, 6 of 9 FDA-approved BsAbs—blinatumomab, amivantamab, teclistamab, mosunetuzumab, epcoritamab, and glofitamab—show low ADA incidence (<3%) (220). However, tebentafusp (gp100×CD3), based on an engineered TCR, exhibits significantly higher immunogenicity, with ADA detected in 29–33% of treated patients.

Other platforms activating T cells demonstrate even greater variability. PRS-343 (HER2×4-1BB, Anticalin-based) induced ADA in 27.8% of patients at doses ≥2.5 mg/kg (221). APVO-414 (PSMA×CD3, ADAPTIR platform) showed ADA in more than 50% of patients, leading to discontinuation of clinical development (222).

AFM13 (CD30×CD16A), the first tetravalent TandAb, also raised immunogenicity concerns. Among 28 patients, 15 developed ADAs—50% of which were neutralizing. This effect was likely linked to the chimeric nature of the CD25-specific scFv domain (223). The impact of TandAb’s tetravalent structure on immunogenicity remains an open question.

Strategies to reduce immunogenicity include antibody humanization, linker optimization, and immunosuppressive premedication. However, none of these fully eliminate ADA risk, underscoring the need for personalized immunogenicity monitoring and management to ensure safe and durable bispecific immunotherapies.

**Figure 4:**
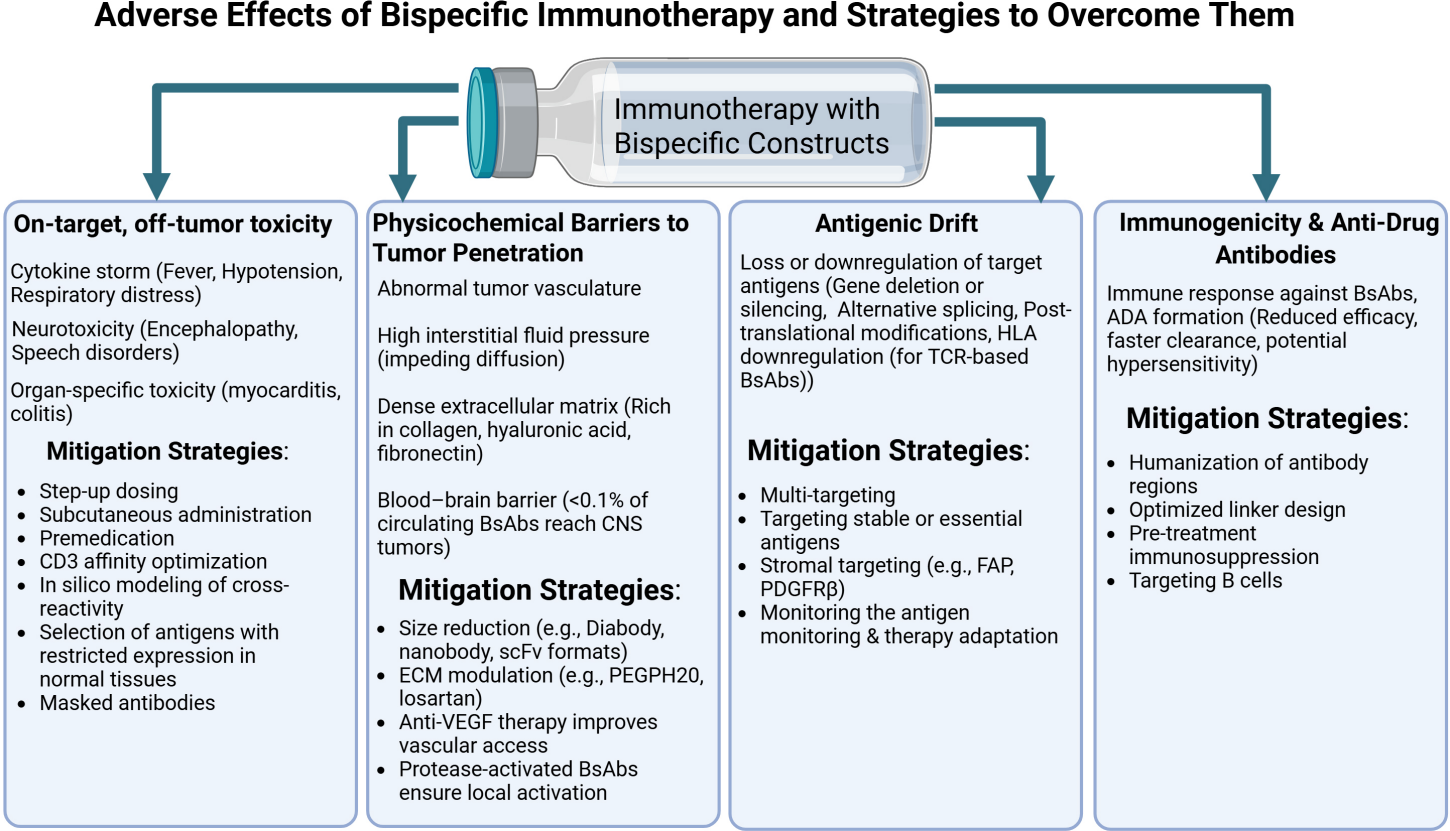
Bispecific antibodies in cancer therapy: side effects, resistance mechanisms and approaches to minimizing complications. Created with Biorender.com.

Further progress in bispecific immunotherapy hinges on a comprehensive assessment of both its therapeutic advantages and the full spectrum of potential complications. Contemporary engineering strategies already demonstrate an ability to balance efficacy with safety (**Figure 4**). Nevertheless, the ultimate selection of platform and treatment regimen must be guided by the tumor’s molecular characteristics, the patient’s immune status, and the specific risk profile of each agent.

The set of described limitations underscores that the development of bispecific therapy is inevitably associated with a number of biological and technological barriers. Off-target toxicity, physicochemical obstacles to tumor tissue penetration, antigenic drift, and immunogenicity with the formation of anti-drug antibodies constitute an interconnected set of challenges that restrict the therapeutic window and reduce the predictability of clinical responses. Overcoming these hurdles requires the integration of engineering, pharmacological, and clinical approaches and will be a defining prerequisite for the further evolution of bispecific constructs and their incorporation into durable and personalized immunotherapy regimens.

## 6 Combinatorial potential of bispecific constructs with other immunotherapeutic approaches

### 6.1 Combinations with cellular therapies

Modern immunotherapeutic strategies increasingly aim to integrate bispecific constructs with genetically modified cell-based therapies to overcome key limitations of each platform—most notably, antigen-negative relapse and effector cell exhaustion. One prominent mechanism of escape in CD19-directed therapy is epitope loss via mutation or alternative splicing of CD19 exon 2 (224). To address this, multispecific constructs have been developed. For example, a trispecific antibody (CD19×CD22×CD3) restored cytotoxicity against CD19^-/low clones *in vitro* and prevented emergence of antigen-negative subpopulations in preclinical B-ALL models (225). Bicistronic CD19/CD22 CAR-T cells achieved durable remissions in 74% of relapsed/refractory DLBCL patients, validating the clinical value of dual targeting (226).

Sequential combinations of BsAbs and autologous CAR-T cells have also proven effective. In an observational series of seven children with relapsed/refractory B-ALL, administration of blinatumomab before leukapheresis reduced tumor burden and achieved complete morphological response in all patients. Post-CAR-T infusion, all remained in remission on day 28, and 57% maintained MRD negativity for ~16 months, underscoring the safety and potential of bridging strategies (227). In multiple myeloma, prior administration of the BCMA×CD3 BsAb teclistamab was shown to bind residual T cells and obscure CAR detection markers without impairing clinical efficacy, highlighting the need for refined monitoring methods (228). In another case, a multi-step regimen for KMT2A-rearranged ALL—including palbociclib, chemotherapy, blinatumomab consolidation, and CD19 CAR-T infusion—led to deep molecular remission without added toxicity (229), suggesting that sequential regimens can extend therapeutic benefit even in poor-risk cytogenetics.

Further support comes from salvage therapy studies following BCMA CAR-T failure. In these settings, talquetamab (GPRC5D×CD3) achieved an overall response rate (ORR) of 79% with complete responses in 39%, while teclistamab (BCMA×CD3) yielded 64% ORR and 32% complete responses—outperforming conventional IMiD/PI/anti-CD38 regimens (230). Multivariate analysis confirmed talquetamab and teclistamab as independent predictors of improved overall survival, including in patients with extramedullary relapse.

Another emerging area is BsAb priming of TCR-T cells. Tebentafusp (gp100-ImmTAC) extended median overall survival in metastatic uveal melanoma to 73 months (119). Single-cell sequencing revealed that tebentafusp reprograms M2 macrophages toward a proinflammatory phenotype, and co-administration of IL-2 further enhanced this effect (231). These findings support the development of TCR-based products targeting antigens like MAGE-A4 and NY-ESO-1, with myeloid cell modulation seen as essential for durable tumor eradication. A highly promising avenue involves NK cell precomplexing with bispecific antibodies, exemplified by AFM13 (CD30×CD16A). In preclinical models, “NK–AFM13” complexes exhibited CAR-like activity and outperformed both unarmed NK cells and AFM13 monotherapy (232). Subsequent studies confirmed this strategy’s potential in CD30-positive hematologic malignancies and justified further clinical testing (233).

Nevertheless, expanding the cytotoxic arsenal raises cumulative toxicity concerns. Talquetamab induced CRS in 74.5–79% of patients, although most events were grade 1 or 2, and grade ≥3 occurred in only 0.7–2.1% of cases; ICANS remained rare (64, 234). These insights underscore the rationale for introducing BsAbs earlier in treatment, when T-cell fitness is still preserved (235, 236).

Future improvements focus on tri- and tetraspecific formats. An optimized CD19/CD22/CD3 “sigma-molecule” provided synergistic T-cell activation at low antigen densities, and reduced CD3 affinity helped lower CRS rates without compromising cytotoxicity (237). Similar dual-targeting constructs (e.g., BCMA + FcRH5, or BCMA + GPRC5D) are in development to overcome BCMA-negative relapse (238). Despite encouraging outcomes, the optimal sequencing of immunomodulatory approaches remains to be fully defined (239). However, pharmacoeconomic models indicate that survival benefits associated with BsAb–cell therapy combinations justify their cost-effectiveness (230).

Going forward, clinical implementation of hybrid strategies should be accompanied by standardized monitoring of cytokine profiles and immune cell subsets, as well as prophylactic use of IL-1/IL-6 inhibitors and early dose escalation protocols. Collectively, the growing body of evidence suggests that rationally designed combinations of bispecific formats with cellular products can significantly expand the therapeutic window of T-cell redirection, offering a pathway toward durable remissions—even in patients with the most treatment-refractory disease.

### 6.2 Combinations with immune checkpoint inhibitors

Bispecific antibodies (BsAbs) form high-affinity immunological synapses between T cells and tumor cells. However, within hours of activation, T cells upregulate inhibitory receptors such as PD-1, TIM-3, LAG-3, and KLRG1, which promotes early functional exhaustion and stimulates immunosuppressive signaling within the tumor microenvironment (240, 241). While checkpoint inhibitors (CPIs) have revolutionized treatment of metastatic melanoma, non-small cell lung cancer (NSCLC), and renal cell carcinoma, their clinical efficacy is limited by the lack of predictive biomarkers, the emergence of both primary and acquired resistance, and high treatment costs (242, 243).

Combining BsAbs with PD-(L)1 inhibitors is therefore being explored as a strategy to both activate and “unblock” T-cell responses. Classical xenograft and 3D tumor spheroid studies have shown that CD3-engaging BsAbs in combination with PD-1 blockade double cytotoxicity and prolong survival compared to monotherapies, while also promoting long-term memory formation (240). Clinical proof-of-concept was demonstrated by complete leukemic eradication in a patient with CD19^+^ leukemia treated with blinatumomab and nivolumab (244).

This principle also underlies triplet-targeting strategies. Preclinical data combining amivantamab (EGFR×c-MET BsAb) with pembrolizumab (anti–PD-1) in NSCLC showed increased infiltration of granzyme B–positive CD8^+^ T cells, expansion of central memory populations, and reduced tumor burden relative to either agent alone (245). These findings supported the launch of the ongoing Phase I/II PolyDamas trial (NCT05908734), evaluating amivantamab plus cetrelimab in metastatic NSCLC. The evolution from bispecific to trispecific and tetraspecific constructs further deepens this concept. For example, the IgTT-4E1-S antibody, targeting PD-L1, EGFR, and 4-1BB, enables selective PD-L1 blockade and conditional 4-1BB activation in EGFR^+^ tumor cells. This induces robust T and NK cell activation without systemic toxicity (246).

Dual targeting of PD-(L)1 and co-stimulatory receptors such as 4-1BB is now moving toward trispecific constructs that integrate checkpoint blockade with localized immune cell activation. The tetraspecific antibody ATG-101 (PD-L1×4-1BB) activates 4-1BB only in PD-L1^+^ cells, converting CPI-refractory tumors to an inflamed phenotype in non-human primates without hepatotoxicity (247). A similar mechanism is seen with PRS-344/S095012, in which an Anticalin module delivers a 4-1BB signal specifically to PD-L1^+^ tumors, eliciting more potent T-cell activation than separate anti–PD-L1 and anti–4-1BB antibodies (248). The scMATCH3 platform is a logical extension of this design. Its lead trispecific molecule, NM21-1480 (PD-L1/4-1BB/albumin), allows conditional 4-1BB co-stimulation, eliminates hepatotoxicity seen with prior agents, and induces tumor regression in xenografts (249). When combined with NM28-2746, a highly selective T-cell engager targeting mesothelin, NM21–1480 enhances T-cell infiltration and suppresses pancreatic tumor growth (250). A further step has been the development of trispecific nanobodies targeting PD-L1, 4-1BB, and NKG2A/TIGIT, which simultaneously activate NK and CD8^+^ T cells, suppressing tumor organoids and xenografts in humanized mouse models (251).

In summary, accumulating preclinical and early clinical evidence indicates that integrating checkpoint inhibitors with bi- and trispecific platforms—particularly those combining PD-(L)1 blockade with targeted 4-1BB agonism—can overcome immune resistance, expand the therapeutic window, and maintain a favorable safety profile. These benefits depend on cytokine monitoring and prompt management of adverse events, underscoring the need for precision immunotherapy design.

### 6.3 Combination with oncolytic viruses

The combination of bispecific constructs with oncolytic viruses (OVs) is emerging as one of the most promising strategies in antitumor immunotherapy. This approach offers several advantages: it bypasses the need for cell-based manufacturing, enables pharmacological delivery of active agents, and has the potential to convert “cold” tumors into immunologically active lesions—an essential step in improving the efficacy of immunotherapy. OVs are a unique platform for modulating the tumor microenvironment. By inducing immunogenic tumor cell lysis and releasing damage-associated molecular patterns (DAMPs), type I interferons, and chemokines (CXCL9/10), they enhance the recruitment of CD8^+^ T cells into the tumor milieu (252). Bispecific T-cell engagers (BsAbs/BiTEs), in turn, potentiate this effect by redirecting activated T cells to tumor antigens, thereby overcoming barriers imposed by heterogeneous antigen expression (253).

Preclinical models have shown that OVs can sensitize tumors to subsequent bispecific antibody therapy. For instance, intratumoral injection of type 3 reovirus into immunocompetent KPC3 pancreatic cancer models triggered IFN responses and CD8^+^ T-cell infiltration. Systemic administration of CD3-specific BsAbs thereafter induced tumor regression and controlled metastases, highlighting the value of OVs as preconditioning agents (252). Additional data come from ICOVIR-15K, an oncolytic adenovirus engineered to express an EGFR-targeted BiTE (cBiTE). The virus retained oncolytic activity and induced sustained T-cell activation and infiltration. Compared to the unmodified virus, ICOVIR-15K-cBiTE significantly enhanced antitumor responses in xenograft models (253).

The adenovirus TILT-321 (Ad5/3-E2F-d24-aMUC1aCD3), expressing a MUC1×CD3 engager, replicated selectively in tumor cells and enabled local engager expression. When combined with allogeneic T cells in ovarian cancer models, TILT-321 increased CD3^+^ infiltration and elicited potent antitumor activity, effectively bypassing the limitations of systemic BiTE delivery in solid tumors (254).

Another example is EnAdenotucirev (EnAd), an adenovirus armed with an EpCAM×CD3 BiTE. Use of a late viral promoter restricted engager expression to replicating tumor cells, enhancing specificity. In patient-derived ascites and pleural fluid, this construct triggered localized activation of CD4^+^ and CD8^+^ T cells and tumor lysis in immunosuppressed environments (255).

A creative approach to overcoming stromal barriers involved an ICOVIR-15K variant expressing a BiTE against CD3 and fibroblast activation protein (FAP). This induced T-cell proliferation and specific cytotoxicity against FAP^+^ cells *in vitro* and *in vivo*, leading to FAP depletion, improved T-cell infiltration, and enhanced antitumor efficacy. In hematologic malignancies such as CD19^+^ lymphomas and acute leukemias, blinatumomab’s short half-life necessitates continuous infusion. To address this, vectors that stably express BiTEs *in vivo* have been developed. An AAV8 vector encoding CD19×CD3 under a liver-specific TBG promoter enabled durable BiTE expression, CD8^+^ T-cell activation, and complete remission with minimal toxicity in B-ALL and DLBCL models (256).

A similar strategy was applied using adenoviral delivery of a B7-H3×CD3 BiTE, targeting a broadly expressed antigen in solid tumors. Local BiTE expression in tumor tissue promoted polyclonal T-cell activation, proliferation, cytokine production, and tumor regression without systemic toxicity (257).

However, the clinical application of viral vectors faces several challenges, including neutralization by pre-existing antibodies and the risk of cytokine release syndrome (CRS). Engineering solutions include PEGylation to reduce phagocytosis, immunogenicity, and extend circulation time (258–260). Local expression of bispecific molecules within the tumor is gaining favor as it achieves high local concentrations of the therapeutic agent with minimal systemic exposure, thus reducing risks such as CRS and cytopenia. Preclinical models using vectors encoding B7-H3×CD3 and EGFR×CD3 BiTEs demonstrated robust CD4^+^/CD8^+^ T-cell activation and localized immune responses without systemic toxicity (257). These findings are reinforced by review articles emphasizing the importance of local engager generation in infected tumors to enhance efficacy while minimizing adverse effects (261). Compared to T-cell–based platforms such as CAR-T or TCR-T, OV-BiTE technologies offer several advantages: they allow off-the-shelf manufacturing, flexible dosing, and multiple routes of administration. Moreover, their ability to inflame cold tumors makes them especially promising for immunologically inert solid tumors. In conclusion, the combination of oncolytic viruses with bispecific constructs represents a synergistic and highly adaptable platform capable of overcoming key barriers in current immunotherapy and expanding the armamentarium for treating resistant and difficult-to-treat cancers.

**Figure 5:**
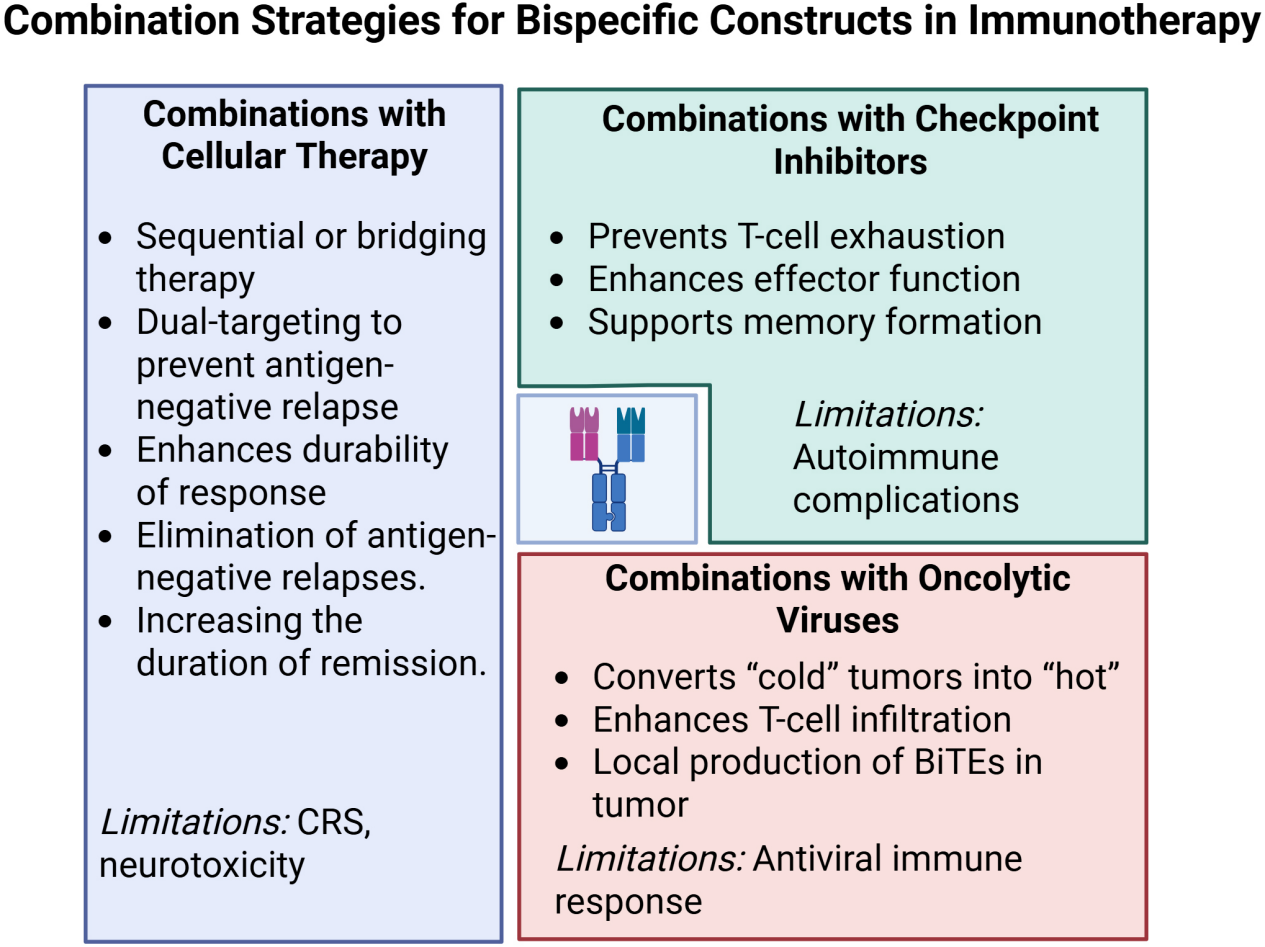
Optimizing cancer immunotherapy: combining bispecific antibodies with cellular, viral, and targeted approaches. Created with Biorender.com.

Thus, integrating bispecific therapies with complementary immunotherapeutic strategies sets the course toward more durable and personalized cancer control (**Figure 5**). Ongoing refinements—from multispecific architectures and locally activatable formats to virus-encoded engagers—continue to widen the therapeutic window while reducing systemic toxicity. Nevertheless, bringing these advances into everyday practice will require harmonized manufacturing standards, predictive biomarkers for efficacy and safety, and adaptive clinical algorithms that account for tumor heterogeneity and immune fitness.

Comparative analysis and examples of combined strategies demonstrate that bispecific constructs hold strong potential for integration with other modern immunotherapeutic approaches. Their combination with cellular technologies helps to overcome antigen-negative relapses and functional exhaustion of effector cells; pairing with immune checkpoint inhibitors enhances antitumor responses by alleviating suppressive mechanisms; and co-application with oncolytic viruses promotes remodeling of the tumor microenvironment and increases its immunogenicity. Taken together, these findings indicate that bispecific immunotherapy may serve as a crucial component of multimodal treatment regimens, providing synergistic effects.

## 7 Promising strategies for the development of multispecific immunotherapy technologies

Modern strategies for the development of multispecific therapeutic agents are aimed at overcoming key limitations related to efficacy and safety through comprehensive optimization of their molecular properties and delivery platforms. A central direction is the refinement of design and engineering, including fine-tuning of binding affinity and the development of tri-, tetra-, and multispecific formats. The efficacy and safety of BsAbs are tightly dependent on the appropriateness of their molecular design. Proper adjustment of affinity for both binding sites can reduce on-target/off-tumor effects while maintaining sufficient cytotoxicity. It has been demonstrated that affinity for CD3 determines the balance between efficacy and safety of anti-HER2/CD3 T-cell–dependent bispecific antibodies: high CD3 affinity increased cytokine release and toxicity, whereas reduced affinity improved tolerability while preserving antitumor activity (262).

Recent studies have shown that the therapeutic window of T-cell–redirecting bispecific antibodies (T-BsAbs) can be expanded by using novel variants of anti-CD3 domains. Using a sequence-based screening platform, humanized anti-CD3 antibodies with distinct epitope specificities and varying levels of T-cell activation were identified; one of these candidates induced sustained tumor cell lysis with minimal cytokine release both *in vitro* and in murine xenograft models, highlighting the promise of next-generation T-BsAbs with improved safety profiles (263). For the bispecific antibody TNB-585 (CD3×PSMA), incorporation of a modified low-affinity anti-CD3 domain resulted in efficient T-cell activation and PSMA-positive tumor eradication *in vitro* and *in vivo*, with reduced cytokine release syndrome compared to high-affinity anti-CD3–based analogues (264). In parallel, engineering approaches targeting Fc domains with partial “knob-into-hole” mutations are being explored to generate alternative constructs (265).

Although available data remain limited, the first clinical trials of low-affinity multispecific constructs, particularly in hematology, have shown encouraging results (266), and definitive conclusions regarding their advantages are expected as more advanced studies are completed. Optimization requires an integrated consideration of CD3 affinity, construct format and geometry, and the choice of appropriate preclinical models. This comprehensive approach will enable the achievement of an optimal balance between efficacy and safety and broaden the therapeutic use of these agents beyond oncology (267).

Tetra- and multispecific formats represent another promising avenue of optimization, allowing the simultaneous blockade of multiple signaling axes and thereby reducing the risk of adaptive resistance. Costimulatory trispecific antibodies, combining binding to a tumor antigen, CD3, and additional receptors (4-1BB, OX40, or CD28), enhance T-cell activation and proliferation, increase metabolic activity, and decrease exhaustion markers. These constructs have demonstrated potent antitumor efficacy in preclinical solid tumor models, supporting their potential for overcoming the limitations of classical bispecific antibodies (268). Tetraspecific antibodies such as FL518 and CRTB6, simultaneously targeting EGFR, HER2, HER3, and VEGF, effectively suppress their respective signaling pathways *in vitro* and *in vivo*, disrupt HER–MET cross-talk, and outperform “two-in-one” and several bispecific constructs across multiple tumor models (269).

At present, several tetraspecific antibodies are undergoing clinical evaluation: a multicenter, open-label phase 1/2 study of GNC-038 (anti-CD19/CD3E/TNFRSF9/PD-L1) in patients with relapsed or refractory diffuse large B-cell lymphoma (NCT05192486); a phase 1/2 study of MDX2001 (anti-c-Met/TROP2/CD3/CD28) in patients with advanced solid tumors (NCT06239194); and a phase 1/2 study of IPH6501 (anti-CD20, 4-1BB, and IL-2Rβ) in patients with relapsed/refractory B-cell non-Hodgkin lymphoma (NCT06088654) (270–272).

Another promising direction in the development of bi- and multispecific constructs is the concept of masked bispecific antibodies that function as prodrugs. The principle of these constructs lies in shielding the binding domains (for example, the CD3-binding scFv) with peptide “masks” or structural elements that block interaction with T cells in the systemic circulation. Antibody activation occurs only within the tumor microenvironment through protease-mediated cleavage, removal of steric hindrance via proteolytic processing, or activation by soluble factors (273). Experimental studies have demonstrated that the toxicity of bispecific constructs can be reduced using prodrug formats in which the anti-CD3 moiety is masked by an autoinhibitory motif and activated exclusively by tumor-associated proteases such as MMP-2, thereby inducing selective T-cell antitumor activity *in vitro* (92). One such construct, Prot-FOLR1-TCB—a protease-activated bispecific antibody with an anti-idiotypic mask on the anti-CD3 domain—regains activity upon linker cleavage by tumor-specific proteases and provides an antitumor effect comparable to its unmasked counterpart. This strategy prevents damage to normal tissues with low FOLR1 expression and has also been validated in mesothelin models, demonstrating enhanced specificity and safety of modified bispecific constructs (8). Similarly, masked scFv T-BsAbs cleaved by collagenase with tumor-specific protease activity showed efficient release of agonistic scFv without undesirable fragmentation, thereby restoring the ability of the masked scFv BsAbs to bind T cells (274).

Although no clinical trials of prodrug-BsAbs per se have yet been registered, the Probody platform (CytomX) has already advanced several masked prodrug antibodies into the clinic (275). These findings support the feasibility of locally activatable antibody strategies, and it is anticipated that similar approaches will soon be adapted for bispecific formats.

An unconventional engineering approach involves the introduction of pH-dependent mutations to improve therapeutic efficacy and safety. The range of pH values in the human body provides a window for designing antibodies sensitive to pH. In the study by Sulea T, Her2-binding antibodies with pH-dependent affinity were tested: binding selectivity and growth inhibition of spheroids were significantly higher in acidic environments compared to physiological pH (276). Although this strategy has thus far been applied primarily to monospecific antibodies (277), its promise suggests that it may also be explored in engineering solutions for multispecific constructs.

Thus, the concept of prodrug-BsAbs is currently at the stage of active preclinical and clinical development, with convincing evidence of reduced systemic toxicity while preserving antitumor activity, making the transition into clinical practice a logical next step.

An important direction with the potential to increase the efficacy and specificity of bispecific construct delivery is the development of novel delivery platforms. Over the past decades, lipid nanoparticles (LNPs) have been actively investigated as a delivery vehicle for immunomodulators, providing protection through encapsulation, surface modification for targeting, stimulus-responsive activation, and reduced nonspecific toxicity (278). This area is highly promising, although the majority of studies remain at the preclinical stage. Two main approaches are being developed: (1) creating a platform for endogenous antibody expression using *in vitro* transcription to generate nucleoside-modified mRNA subsequently encapsulated in LNPs, thereby transforming the body’s own cells into a production factory; and (2) using bispecific antibodies as targeting moieties to guide the delivery of LNP-encapsulated mRNA encoding tumor-specific proteins directly to tumors.

As an example of the first approach, an mRNA–LNP platform has been developed for *in vivo* expression of a bispecific antibody (XA-1) that simultaneously blocks the immune checkpoints PD-1 and PD-L1. This strategy demonstrated superior efficacy compared with direct administration of XA-1 protein, providing improved pharmacokinetics and more pronounced antitumor activity in murine models (279). Another example involves delivery of mRNA encoding the bispecific antibody B7H3×CD3 (BiTE) encapsulated in ionizable LNPs, which enabled high *in vivo* protein expression and significantly extended its half-life. LNPs showed high transfection efficiency and tropism for the liver and spleen, leading to the production of high concentrations of BiTE. A single injection of this construct produced sustained antitumor efficacy against hematological malignancies and melanoma in experimental settings, underscoring the clinical potential of this approach (280). Based on preclinical data showing that BNT142—an mRNA–LNP encoding a bispecific antibody targeting CD3 and the tumor antigen CLDN6—achieved durable production of functional antibody in animals and complete regression of CLDN6-positive tumors, a phase 1/2 clinical program evaluating BNT142 in patients with advanced solid tumors has been initiated (NCT05262530) (281). Similarly, the standard HER2-CD3-Fc structure has been delivered as an mRNA–LNP, demonstrating high binding affinity and the ability to induce potent, specific T-cell cytotoxicity against HER2-positive tumor cells *in vitro* and *in vivo* (282). An mRNA–LNP platform for hepatic expression of a bispecific IFN-α/anti-GPC3 protein showed significant antitumor activity against GPC3-positive hepatocellular carcinoma by enhancing CD8^+^ T-cell infiltration and synergizing with anti-PD-1 therapy. Importantly, this strategy provided a broad therapeutic window, with a maximum tolerated dose 40-fold higher than the minimal effective dose (283). A further step has been the combination of two bispecific antibodies encoded by separate mRNAs in LNPs, with complementary functions: anti-EGFR×CD3 for T-cell activation and anti-PD-L1×4-1BB with extended half-life for co-stimulation. This approach achieved sustained antibody expression with programmable pharmacokinetics, resulting in significant regression of EGFR-positive tumors without associated toxicity in preclinical models (284).

As an example of the second approach, a strategy was developed in which bispecific antibodies were used for targeted delivery of nucleic acid–based therapeutics to the surface of tumor cells with high expression of glucose-regulated protein 78 (GRP78). Functional analysis demonstrated the advantages of this approach in terms of high antigen-binding affinity, tumor selectivity, improved cellular uptake, and efficient gene expression (285). An innovative method of nonchemical conjugation of LNPs with bispecific antibodies was also developed, in which one antibody domain binds an epitope of hemagglutinin embedded in the LNP, while the other targets membrane proteins such as PD-L1, CD4, or CD5. This strategy significantly enhanced specificity and transfection efficiency both *in vivo* and ex vivo, providing a simple and universal method for targeted mRNA delivery (286). Another technology enables rapid development of targeted mRNA-based therapeutics by replacing the targeting domain in a BsAb. This strategy demonstrated efficient delivery of mRNA–LNPs beyond the liver, targeting cells positive for epidermal growth factor receptor (EGFR) and folate hydrolase 1 (PSMA) *in vitro* and *in vivo* (287).

**Figure 6:**
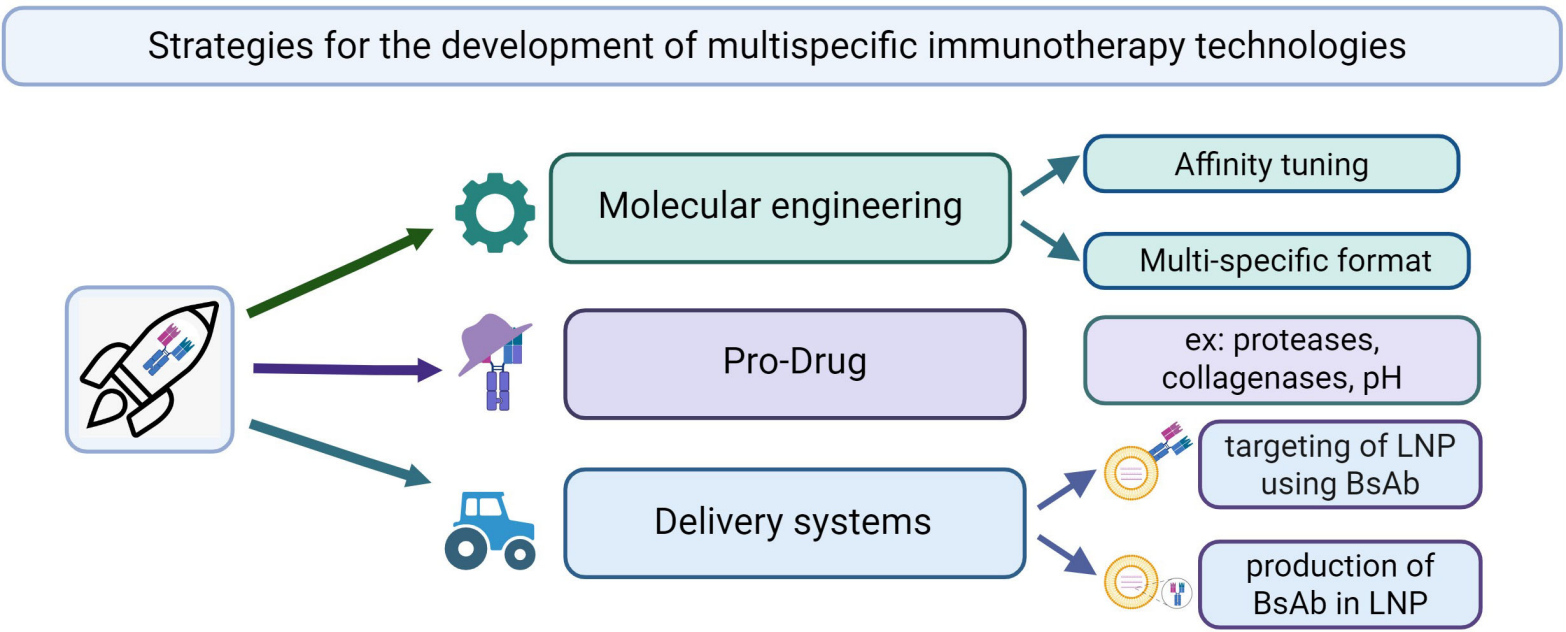
Strategies for the development of multispecific immunotherapy technologies. Created with Biorender.com.

A summarized scheme of strategies for the development of multimer-based technologies is presented in **Figure 6**.

Thus, the integration of molecular engineering strategies with delivery platforms establishes a new paradigmatic approach in multispecific immunotherapy, in which the simultaneous optimization of affinity, valency, activation potential, and targeting enables the overcoming of fundamental limitations of traditional modalities. The body of preclinical evidence together with initial clinical results indicates that the synergistic combination of these technologies will open the way to the development of personalized therapeutic regimens with controllable activation and improved safety profiles, which in the future may substantially transform approaches to cancer treatment.

## 8 Conclusion

Bispecific immunotherapy represents one of the most rapidly advancing and promising approaches in modern oncology. Its foundation lies in the ability to simultaneously recognize two molecular targets, thereby substantially expanding the functional capabilities of immune agents through enhanced specificity, efficacy, and resistance to tumor immune evasion. This review demonstrates that over the past two decades, bispecific constructs have evolved from experimental, unstable, and often toxic molecules into clinically validated therapeutics with controlled pharmacokinetics and high therapeutic indices.

The analysis of current trends in the development of multispecific immunotherapy suggests that the era of monospecific therapeutic solutions in oncoimmunology is coming to an end. The future of the field lies not in the creation of a single “miracle drug,” but in the development of complex, adaptive, and personalized therapeutic ecosystems in which multispecific antibodies and receptors (TCRs) will play a central, though not exclusive, role. The key paradigmatic shift is likely to be the transition from simple combination of approaches to their deep integration into unified, logically structured therapeutic frameworks. This integration should unfold at several interconnected levels.

The first and fundamental level is target selection. Current practice in antigen choice for targeted therapy still largely resembles “shooting sparrows with a cannon”: a limited set of targets (such as CD19, HER2, EGFR) is applied to highly heterogeneous patient populations. However, it is becoming increasingly evident that each patient’s tumor possesses a unique antigenic landscape, shaped both by genomic instability and the tumor microenvironment. Accumulating data highlight the necessity of introducing high-throughput methods for comprehensive tumor antigen profiling into clinical practice. Such in-depth analysis will allow the transition from a “one antigen fits all” strategy to an “optimal antigen for each” approach. This, in turn, will pave the way for personalized selection of bispecific antibodies or TCR-containing constructs, thereby increasing therapeutic specificity and minimizing the risk of on-target/off-tumor toxicity. Nevertheless, target selection alone, without accurate prediction of its consequences, demands a rigorous safety evaluation system. Here, the second critical element of the future ecosystem comes into focus—the integration of in silico toxicity prediction methods.

It is reasonable to assume that the development of each new multispecific agent should be accompanied by comprehensive computational modeling. This entails the creation of advanced physiologically based pharmacokinetic (PBPK) and pharmacodynamic (PBPK-PD) models that account not only for drug distribution in the body but also for its interactions with the immune system. Modern machine learning algorithms, trained on extensive datasets of antigen expression in normal tissues, are already showing the capacity to predict potential cross-reactivity and related organ-specific toxicities with high accuracy. This will not only save substantial resources but also prevent failures in late-stage clinical trials caused by unforeseen toxicities.

The third strategic level of integration is the synergy of multispecific constructs with cellular immunotherapy, particularly CAR-T and TCR-T cells. At present, these modalities are often perceived as competing; however, growing evidence points toward their complementary nature. Circulating multispecific antibodies could potentially act as “pathfinders,” preparing the tumor niche for subsequent cellular attack. Designing the conditions for such combinations is a systems biology challenge that requires precise dosing, sequencing of administration, and real-time biomarker monitoring.

A fourth essential component of the emerging paradigm is the “priming step”—the transformation of immunologically “cold” tumors into “hot” ones. Multispecific therapy demonstrates maximal efficacy against tumors with immune infiltration, necessitating microenvironmental reprogramming. The use of immune checkpoint inhibitors (anti-PD-1, anti-CTLA-4) prior to or in parallel with bispecific antibody administration represents a rational therapeutic strategy, as these drugs relieve immunosuppressive signals from tumor-infiltrating lymphocytes, creating favorable conditions for subsequent activation by bispecific constructs. Equally promising are oncolytic viruses, which serve a dual function: direct lysis of tumor cells and generation of strong inflammatory signals that attract immune cells to the tumor niche. The release of tumor antigens upon viral lysis, combined with an inflammatory microenvironment, establishes optimal conditions for bispecific therapy.

In the context of preclinical research, the rapidly expanding number of promising molecules underscores the need for more advanced preclinical models. Traditional 2D cell cultures and xenograft models in immunodeficient mice have limited predictive capacity for complex immune interactions and the influence of three-dimensional tumor architecture. In this regard, advances in 3D culture systems, particularly organoids and organotypic tumor slices, are of great interest. These models preserve tumor heterogeneity and its microenvironment and enable the study of immune cell infiltration and activity under conditions closely resembling *in vivo*. The use of such 3D models in high-throughput formats creates opportunities for large-scale screening of combinations involving multispecific antibodies, cellular therapies, and immunomodulators on patient-derived material. This represents a practical implementation of personalized medicine principles at the preclinical stage.

In conclusion, the future of multispecific immunotherapy lies in the synergistic convergence of diverse technological platforms. The optimal therapeutic strategy will likely involve a sequential process: deep multi-omic profiling of the patient’s tumor, computational design of therapeutic agents with predicted minimal toxicity, validation in authentic 3D models, and the administration of a cascade of complementary interventions. Such an approach, requiring interdisciplinary collaboration among bioinformaticians, immunologists, engineers, and clinicians, opens the way to achieving a qualitatively new level of control over cancer.

## References

[1] Shan KS Musleh Ud Din S Dalal S Gonzalez T Dalal M Ferraro P. Bispecific antibodies in solid tumors: advances and challenges. Int J Mol Sci. (2025) 26:5838. doi: 10.3390/ijms26125838, PMID: 40565299 PMC12192982

[2] Labrijn AF Janmaat ML Reichert JM Parren PWHI. Bispecific antibodies: a mechanistic review of the pipeline. Nat Rev Drug Discov. (2019) 18:585–608. doi: 10.1038/s41573-019-0028-1, PMID: 31175342

[3] Herrera M Pretelli G Desai J Garralda E Siu LL Steiner TM. Bispecific antibodies: advancing precision oncology. Trends Cancer. (2024) 10:893–919. doi: 10.1016/j.trecan.2024.07.002, PMID: 39214782

[4] Wu Y Yi M Zhu S Wang H Wu K. Recent advances and challenges of bispecific antibodies in solid tumors. Exp Hematol Oncol. (2021) 10:56. doi: 10.1186/s40164-021-00250-1, PMID: 34922633 PMC8684149

[5] Qin X Ning W Liu H Liu X Luo W Xia N. Stepping forward: T-cell redirecting bispecific antibodies in cancer therapy. Acta Pharm Sin B. (2024) 14:2361–77. doi: 10.1016/j.apsb.2024.03.027, PMID: 38828136 PMC11143529

[6] Cho BC Lu S Felip E Spira AI Girard N Lee JS. Amivantamab plus lazertinib in previously untreated EGFR-mutated advanced NSCLC. N Engl J Med. (2024) 391:1486–98. doi: 10.1056/NEJMoa2403614, PMID: 38924756

[7] Harding JJ Fan J Oh DY Choi HJ Kim JW Chang HM. Zanidatamab for HER2-amplified, unresectable, locally advanced or metastatic biliary tract cancer (HERIZON-BTC-01): a multicentre, single-arm, phase 2b study. Lancet Oncol. (2023) 24:772–82. doi: 10.1016/S1470-2045(23)00242-5, PMID: 37276871

[8] Geiger M Stubenrauch KG Sam J Richter WF Jordan G Eckmann J. Protease-activation using anti-idiotypic masks enables tumor specificity of a folate receptor 1-T cell bispecific antibody. Nat Commun. (2020) 11:3196. doi: 10.1038/s41467-020-16838-w, PMID: 32581215 PMC7314773

[9] Sun Y Yu X Wang X Yuan K Wang G Hu L. Bispecific antibodies in cancer therapy: Target selection and regulatory requirements. Acta Pharm Sin B. (2023) 13:3583–97. doi: 10.1016/j.apsb.2023.05.023, PMID: 37719370 PMC10501874

[10] Ojemolon PE Kalidindi S Ahlborn TA Aihie OP Awoyomi MI. Cytokine release syndrome following blinatumomab therapy. Cureus. (2022) 14:e21583. doi: 10.7759/cureus.21583, PMID: 35228941 PMC8867529

[11] Schaefer W Regula JT Bähner M Schanzer J Croasdale R Dürr H. Immunoglobulin domain crossover as a generic approach for the production of bispecific IgG antibodies. Proc Natl Acad Sci U S A. (2011) 108:11187–92. doi: 10.1073/pnas.1019002108, PMID: 21690412 PMC3131342

[12] Mandrup OA Ong SC Lykkemark S Dinesen A Rudnik-Jansen I Dagnæs-Hansen NF. Programmable half-life and anti-tumour effects of bispecific T-cell engager-albumin fusions with tuned FcRn affinity. Commun Biol. (2021) 4:310. doi: 10.1038/s42003-021-01790-2, PMID: 33686177 PMC7940400

[13] Zeng X Zhang H Guo J Yang D Zhu Y Liu N. A novel bispecific T-cell engager using the ligand-target csGRP78 against acute myeloid leukemia. Cell Mol Life Sci. (2024) 81:371. doi: 10.1007/s00018-024-05410-0, PMID: 39196413 PMC11358366

[14] Reusch U Burkhardt C Fucek I Le Gall F Le Gall M Hoffmann K. A novel tetravalent bispecific TandAb (CD30/CD16A) efficiently recruits NK cells for the lysis of CD30+ tumor cells. MAbs. (2014) 6:728–39. doi: 10.4161/mabs.28591, PMID: 24670809 PMC4011917

[15] Stapleton NM Einarsdóttir HK Stemerding AM Vidarsson G. The multiple facets of FcRn in immunity. Immunol Rev. (2015) 268:253–68. doi: 10.1111/imr.12331, PMID: 26497526

[16] Zhou F Ben Y Jiang H Tan S Mu G Zha Z. A novel dual-fc bispecific antibody with enhanced fc effector function. Biochemistry. (2024) 63:958–68. doi: 10.1021/acs.biochem.3c00481, PMID: 38426700 PMC11025548

[17] Wang Q Chen Y Park J Liu X Hu Y Wang T. Design and production of bispecific antibodies. Antibodies (Basel). (2019) 8:43. doi: 10.3390/antib8030043, PMID: 31544849 PMC6783844

[18] Vugmeyster Y Xu X Theil FP Khawli LA Leach MW. Pharmacokinetics and toxicology of therapeutic proteins: Advances and challenges. World J Biol Chem. (2012) 3:73–92. doi: 10.4331/wjbc.v3.i4.73, PMID: 22558487 PMC3342576

[19] Mester S Evers M Meyer S Nilsen J Greiff V Sandlie I. Extended plasma half-life of albumin-binding domain fused human IgA upon pH-dependent albumin engagement of human FcRn *in vitro* and *in vivo* . MAbs. (2021) 13:1893888. doi: 10.1080/19420862.2021.1893888, PMID: 33691596 PMC7954421

[20] Klein C Schaefer W Regula JT. The use of CrossMAb technology for the generation of bi- and multispecific antibodies. MAbs. (2016) 8:1010–20. doi: 10.1080/19420862.2016.1197457, PMID: 27285945 PMC4968094

[21] de Jong RN Beurskens FJ Verploegen S Strumane K van Kampen MD Voorhorst M. A novel platform for the potentiation of therapeutic antibodies based on antigen-dependent formation of igG hexamers at the cell surface. PLoS Biol. (2016) 14:e1002344. doi: 10.1371/journal.pbio.1002344, PMID: 26736041 PMC4703389

[22] Vallera DA Felices M McElmurry R McCullar V Zhou X Schmohl JU. IL15 trispecific killer engagers (TriKE) make natural killer cells specific to CD33+ Targets while also inducing persistence, *in vivo* expansion, and enhanced function. Clin Cancer Res. (2016) 22:3440–50. doi: 10.1158/1078-0432.CCR-15-2710, PMID: 26847056 PMC4947440

[23] Sarhan D Brandt L Felices M Guldevall K Lenvik T Hinderlie P. 161533 TriKE stimulates NK-cell function to overcome myeloid-derived suppressor cells in MDS. Blood Adv. (2018) 2:1459–69. doi: 10.1182/bloodadvances.2017012369, PMID: 29941459 PMC6020813

[24] Wang C Vemulapalli B Cao M Gadre D Wang J Hunter A. A systematic approach for analysis and characterization of mispairing in bispecific antibodies with asymmetric architecture. MAbs. (2018) 10:1226–35. doi: 10.1080/19420862.2018.1511198, PMID: 30153083 PMC6284573

[25] Drent E Themeli M Poels R de Jong-Korlaar R Yuan H de Bruijn J. A rational strategy for reducing on-target off-tumor effects of CD38-chimeric antigen receptors by affinity optimization. Mol Ther. (2017) 25:1946–58. doi: 10.1016/j.ymthe.2017.04.024, PMID: 28506593 PMC5542711

[26] Igawa T Mimoto F Hattori K. pH-dependent antigen-binding antibodies as a novel therapeutic modality. Biochim Biophys Acta. (2014) 1844:1943–50. doi: 10.1016/j.bbapap.2014.08.003, PMID: 25125373

[27] Liu Y Lee AG Nguyen AW Maynard JA. An antibody Fc engineered for conditional antibody-dependent cellular cytotoxicity at the low tumor microenvironment pH. J Biol Chem. (2022) 298:101798. doi: 10.1016/j.jbc.2022.101798, PMID: 35248534 PMC9006656

[28] Tansi FL Rüger R Böhm C Steiniger F Kontermann RE Teichgraeber UK. Activatable bispecific liposomes bearing fibroblast activation protein directed single chain fragment/Trastuzumab deliver encapsulated cargo into the nuclei of tumor cells and the tumor microenvironment simultaneously. Acta Biomater. (2017) 54:281–93. doi: 10.1016/j.actbio.2017.03.033, PMID: 28347861

[29] Bargou R Leo E Zugmaier G Klinger M Goebeler M Knop S. Tumor regression in cancer patients by very low doses of a T cell-engaging antibody. Science. (2008) 321:974–7. doi: 10.1126/science.1158545, PMID: 18703743

[30] Haber L Olson K Kelly MP Crawford A DiLillo DJ Tavaré R. Generation of T-cell-redirecting bispecific antibodies with differentiated profiles of cytokine release and biodistribution by CD3 affinity tuning. Sci Rep. (2021) 11:14397. doi: 10.1038/s41598-021-93842-0, PMID: 34257348 PMC8277787

[31] Pahl JHW Koch J Götz JJ Arnold A Reusch U Gantke T. CD16A activation of NK cells promotes NK cell proliferation and memory-like cytotoxicity against cancer cells. Cancer Immunol Res. (2018) 6:517–27. doi: 10.1158/2326-6066.CIR-17-0550, PMID: 29514797

[32] Felices M Lenvik TR Kodal B Lenvik AJ Hinderlie P Bendzick LE. Potent cytolytic activity and specific IL15 delivery in a second-generation trispecific killer engager. Cancer Immunol Res. (2020) 8:1139–49. doi: 10.1158/2326-6066.CIR-19-0837, PMID: 32661096 PMC7484162

[33] Kuhné MR Lin J Yablonski D Mollenauer MN Ehrlich LI Huppa J. Linker for activation of T cells, zeta-associated protein-70, and Src homology 2 domain-containing leukocyte protein-76 are required for TCR-induced microtubule-organizing center polarization. J Immunol. (2003) 171:860–6. doi: 10.4049/jimmunol.171.2.860, PMID: 12847255

[34] Zeng L Palaia I Šarić A Su X. PLCγ1 promotes phase separation of T cell signaling components. J Cell Biol. (2021) 220:e202009154. doi: 10.1083/jcb.202009154, PMID: 33929486 PMC8094118

[35] Xiao X Cheng Y Zheng X Fang Y Zhang Y Sun R. Bispecific NK-cell engager targeting BCMA elicits stronger antitumor effects and produces less proinflammatory cytokines than T-cell engager. Front Immunol. (2023) 14:1113303. doi: 10.3389/fimmu.2023.1113303, PMID: 37114050 PMC10126364

[36] Ruan DY Wei XL Liu FR Hu XC Zhang J Ji DM. The first-in-class bispecific antibody IBI318 (LY3434172) targeting PD-1 and PD-L1 in patients with advanced tumors: a phase Ia/Ib study. J Hematol Oncol. (2024) 17:118. doi: 10.1186/s13045-024-01644-4, PMID: 39614368 PMC11606118

[37] Berezhnoy A Sumrow BJ Stahl K Shah K Liu D Li J. Development and preliminary clinical activity of PD-1-guided CTLA-4 blocking bispecific DART molecule. Cell Rep Med. (2020) 1:100163. doi: 10.1016/j.xcrm.2020.100163, PMID: 33377134 PMC7762776

[38] Schram AM Odintsov I Espinosa-Cotton M Khodos I Sisso WJ Mattar MS. Zenocutuzumab, a HER2xHER3 bispecific antibody, is effective therapy for tumors driven by NRG1 gene rearrangements. Cancer Discov. (2022) 12:1233–47. doi: 10.1158/2159-8290.CD-21-1119, PMID: 35135829 PMC9394398

[39] Park K Haura EB Leighl NB Mitchell P Shu CA Girard N. Amivantamab in EGFR exon 20 insertion-mutated non-small-cell lung cancer progressing on platinum chemotherapy: initial results from the CHRYSALIS phase I study. J Clin Oncol. (2021) 39:3391–402. doi: 10.1200/JCO.21.00662, PMID: 34339292 PMC8791812

[40] Strauss J Heery CR Schlom J Madan RA Cao L Kang Z. Phase I trial of M7824 (MSB0011359C), a bifunctional fusion protein targeting PD-L1 and TGFβ, in advanced solid tumors. Clin Cancer Res. (2018) 24:1287–95. doi: 10.1158/1078-0432.CCR-17-2653, PMID: 29298798 PMC7985967

[41] Zheng S Shen F Jones B Fink D Geist B Nnane I. Characterization of concurrent target suppression by JNJ-61178104, a bispecific antibody against human tumor necrosis factor and interleukin-17A. MAbs. (2020) 12:1770018. doi: 10.1080/19420862.2020.1770018, PMID: 32544369 PMC7531573

[42] Kasaian MT Marquette K Fish S DeClercq C Agostinelli R Cook TA. An IL-4/IL-13 dual antagonist reduces lung inflammation, airway hyperresponsiveness, and IgE production in mice. Am J Respir Cell Mol Biol. (2013) 49:37–46. doi: 10.1165/rcmb.2012-0500OC, PMID: 23449738

[43] Moran A Pavord ID. Anti-IL-4/IL-13 for the treatment of asthma: the story so far. Expert Opin Biol Ther. (2020) 20:283–94. doi: 10.1080/14712598.2020.1714027, PMID: 31914819

[44] Nikkhoi SK Li G Eleya S Yang G Vandavasi VG Hatefi A. Bispecific killer cell engager with high affinity and specificity toward CD16a on NK cells for cancer immunotherapy. Front Immunol. (2023) 13:1039969. doi: 10.3389/fimmu.2022.1039969, PMID: 36685519 PMC9852913

[45] Rothe A Sasse S Topp MS Eichenauer DA Hummel H Reiners KS. A phase 1 study of the bispecific anti-CD30/CD16A antibody construct AFM13 in patients with relapsed or refractory Hodgkin lymphoma. Blood. (2015) 125:4024–31. doi: 10.1182/blood-2014-12-614636, PMID: 25887777 PMC4528081

[46] Wingert S Reusch U Knackmuss S Kluge M Damrat M Pahl J. Preclinical evaluation of AFM24, a novel CD16A-specific innate immune cell engager targeting EGFR-positive tumors. MAbs. (2021) 13:1950264. doi: 10.1080/19420862.2021.1950264, PMID: 34325617 PMC8331026

[47] Rahmani S Yazdanpanah N Rezaei N. Natural killer cells and acute myeloid leukemia: promises and challenges. Cancer Immunol Immunother. (2022) 71:2849–67. doi: 10.1007/s00262-022-03217-1, PMID: 35639116 PMC10991240

[48] Vantourout P Willcox C Turner A Swanson CM Haque Y Sobolev O. Immunological visibility: posttranscriptional regulation of human NKG2D ligands by the EGF receptor pathway. Sci Transl Med. (2014) 6:231ra49. doi: 10.1126/scitranslmed.3007579, PMID: 24718859 PMC3998197

[49] Oberg HH Peipp M Kellner C Sebens S Krause S Petrick D. Novel bispecific antibodies increase γδ T-cell cytotoxicity against pancreatic cancer cells. Cancer Res. (2014) 74:1349–60. doi: 10.1158/0008-5472.CAN-13-0675, PMID: 24448235

[50] Liu J Wu M Yang Y Wang Z He S Tian X. γδ T cells and the PD-1/PD-L1 axis: a love-hate relationship in the tumor microenvironment. J Transl Med. (2024) 22:553. doi: 10.1186/s12967-024-05327-z, PMID: 38858763 PMC11163710

[51] Tseng D Volkmer JP Willingham SB Contreras-Trujillo H Fathman JW Fernhoff NB. Anti-CD47 antibody-mediated phagocytosis of cancer by macrophages primes an effective antitumor T-cell response. Proc Natl Acad Sci U S A. (2013) 110:11103–8. doi: 10.1073/pnas.1305569110, PMID: 23690610 PMC3703977

[52] Dheilly E Moine V Broyer L Salgado-Pires S Johnson Z Papaioannou A. Selective blockade of the ubiquitous checkpoint receptor CD47 is enabled by dual-targeting bispecific antibodies. Mol Ther. (2017) 25:523–33. doi: 10.1016/j.ymthe.2016.11.006, PMID: 28153099 PMC5368402

[53] Zhang B Li W Fan D Tian W Zhou J Ji Z. Advances in the study of CD47-based bispecific antibody in cancer immunotherapy. Immunology. (2022) 167:15–27. doi: 10.1111/imm.13498, PMID: 35575882

[54] Li M Li M Yang Y Liu Y Xie H Yu Q. Remodeling tumor immune microenvironment via targeted blockade of PI3K-γ and CSF-1/CSF-1R pathways in tumor associated macrophages for pancreatic cancer therapy. J Control Release. (2020) 321:23–35. doi: 10.1016/j.jconrel.2020.02.011, PMID: 32035193

[55] Tullett KM Leal Rojas IM Minoda Y Tan PS Zhang JG Smith C. Targeting CLEC9A delivers antigen to human CD141^+^ DC for CD4^+^ and CD8^+^T cell recognition. JCI Insight. (2016) 1:e87102. doi: 10.1172/jci.insight.87102, PMID: 27699265 PMC5033826

[56] Salomon R Rotem H Katzenelenbogen Y Weiner A Cohen Saban N Feferman T. Bispecific antibodies increase the therapeutic window of CD40 agonists through selective dendritic cell targeting. Nat Cancer. (2022) 3:287–302. doi: 10.1038/s43018-022-00329-6, PMID: 35190724

[57] Staerz UD Bevan MJ. Hybrid hybridoma producing a bispecific monoclonal antibody that can focus effector T-cell activity. Proc Natl Acad Sci U S A. (1986) 83:1453–7. doi: 10.1073/pnas.83.5.1453, PMID: 2869486 PMC323094

[58] Topp MS Gökbuget N Stein AS Zugmaier G O’Brien S Bargou RC. Safety and activity of blinatumomab for adult patients with relapsed or refractory B-precursor acute lymphoblastic leukaemia: a multicentre, single-arm, phase 2 study. Lancet Oncol. (2015) 16:57–66. doi: 10.1016/S1470-2045(14)71170-2, PMID: 25524800

[59] Kantarjian H Stein A Gökbuget N Fielding AK Schuh AC Ribera JM. Blinatumomab versus chemotherapy for advanced acute lymphoblastic leukemia. N Engl J Med. (2017) 376:836–47. doi: 10.1056/NEJMoa1609783, PMID: 28249141 PMC5881572

[60] Budde LE Sehn LH Matasar M Schuster SJ Assouline S Giri P. Safety and efficacy of mosunetuzumab, a bispecific antibody, in patients with relapsed or refractory follicular lymphoma: a single-arm, multicentre, phase 2 study. Lancet Oncol. (2022) 23:1055–65. doi: 10.1016/S1470-2045(22)00335-7, PMID: 35803286

[61] Moreau P Garfall AL van de Donk NWCJ Nahi H San-Miguel JF Oriol A. Teclistamab in relapsed or refractory multiple myeloma. N Engl J Med. (2022) 387:495–505. doi: 10.1056/NEJMoa2203478, PMID: 35661166 PMC10587778

[62] Thieblemont C Phillips T Ghesquieres H Cheah CY Clausen MR Cunningham D. Epcoritamab, a novel, subcutaneous CD3xCD20 bispecific T-cell-engaging antibody, in relapsed or refractory large B-cell lymphoma: dose expansion in a phase I/II trial. J Clin Oncol. (2023) 41:2238–47. doi: 10.1200/JCO.22.01725, PMID: 36548927 PMC10115554

[63] Lesokhin AM Tomasson MH Arnulf B Bahlis NJ Miles Prince H Niesvizky R. Elranatamab in relapsed or refractory multiple myeloma: phase 2 MagnetisMM-3 trial results. Nat Med. (2023) 29:2259–67. doi: 10.1038/s41591-023-02528-9, PMID: 37582952 PMC10504075

[64] Chari A Minnema MC Berdeja JG Oriol A van de Donk NWCJ Rodríguez-Otero P. Talquetamab, a T-cell-redirecting GPRC5D bispecific antibody for multiple myeloma. N Engl J Med. (2022) 387:2232–44. doi: 10.1056/NEJMoa2204591, PMID: 36507686

[65] Dickinson MJ Carlo-Stella C Morschhauser F Bachy E Corradini P Iacoboni G. Glofitamab for relapsed or refractory diffuse large B-cell lymphoma. N Engl J Med. (2022) 387:2220–31. doi: 10.1056/NEJMoa2206913, PMID: 36507690

[66] Paz-Ares L Champiat S Lai WV Izumi H Govindan R Boyer M. Tarlatamab, a first-in-class DLL3-targeted bispecific T-cell engager, in recurrent small-cell lung cancer: an open-label, phase I study. J Clin Oncol. (2023) 41:2893–903. doi: 10.1200/JCO.22.02823, PMID: 36689692 PMC10414718

[67] Yun J Lee SH Kim SY Jeong SY Kim JH Pyo KH. Antitumor activity of amivantamab (JNJ-61186372), an EGFR-MET bispecific antibody, in diverse models of EGFR exon 20 insertion-driven NSCLC. Cancer Discov. (2020) 10:1194–209. doi: 10.1158/2159-8290.CD-20-0116, PMID: 32414908

[68] Oldenburg J Mahlangu JN Kim B Schmitt C Callaghan MU Young G. Emicizumab prophylaxis in hemophilia A with inhibitors. N Engl J Med. (2017) 377:809–18. doi: 10.1056/NEJMoa1703068, PMID: 28691557

[69] Meric-Bernstam F Beeram M Hamilton E Oh DY Hanna DL Kang YK. Zanidatamab, a novel bispecific antibody, for the treatment of locally advanced or metastatic HER2-expressing or HER2-amplified cancers: a phase 1, dose-escalation and expansion study. Lancet Oncol. (2022) 23:1558–70. doi: 10.1016/S1470-2045(22)00621-0, PMID: 36400106

[70] Vaishampayan UN Thakur A Chen W Deol A Patel M Dobson K. Phase II trial of pembrolizumab and anti-CD3 x anti-HER2 bispecific antibody-armed activated T cells in metastatic castration-resistant prostate cancer. Clin Cancer Res. (2023) 29:122–33. doi: 10.1158/1078-0432.CCR-22-1601, PMID: 36255393 PMC9812860

[71] Keam SJ. Zolbetuximab: first approval. Drugs. (2024) 84:977–83. doi: 10.1007/s40265-024-02056-x, PMID: 38967717

[72] Schram AM Goto K Kim DW Macarulla T Hollebecque A O’Reilly EM. Efficacy of zenocutuzumab in NRG1 fusion-positive cancer. N Engl J Med. (2025) 392:566–76. doi: 10.1056/NEJMoa2405008, PMID: 39908431 PMC11878197

[73] Demaria O Habif G Vetizou M Gauthier L Remark R Chiossone L. A tetraspecific engager armed with a non-alpha IL-2 variant harnesses natural killer cells against B cell non-Hodgkin lymphoma. Sci Immunol. (2024) 9:eadp3720. doi: 10.1126/sciimmunol.adp3720, PMID: 39546590

[74] Esfandiari A Cassidy S Webster R.M. Bispecific antibodies in oncology. Nat Rev Drug Discov. (2022) 21:411–2. doi: 10.1038/d41573-022-00040-2, PMID: 35246638

[75] Dahan R Reiter Y. T-cell-receptor-like antibodies - generation, function and applications. Expert Rev Mol Med. (2012) 14:e6. doi: 10.1017/erm.2012.2, PMID: 22361332

[76] Duan Z Ho M. Targeting the cancer neoantigens p53 and KRAS with TCR mimic antibodies. Antib Ther. (2021) 4:208–11. doi: 10.1093/abt/tbab021, PMID: 34661061 PMC8515948

[77] Garber K. Driving T-cell immunotherapy to solid tumors. Nat Biotechnol. (2018) 36:215–9. doi: 10.1038/nbt.4090, PMID: 29509745

[78] Linette GP Stadtmauer EA Maus MV Rapoport AP Levine BL Emery L. Cardiovascular toxicity and titin cross-reactivity of affinity-enhanced T cells in myeloma and melanoma. Blood. (2013) 122:863–71. doi: 10.1182/blood-2013-03-490565, PMID: 23770775 PMC3743463

[79] Amir AL van der Steen DM Hagedoorn RS Kester MG van Bergen CA Drijfhout JW. Allo-HLA-reactive T cells inducing graft-versus-host disease are single peptide specific. Blood. (2011) 118:6733–42. doi: 10.1182/blood-2011-05-354787, PMID: 21972290

[80] Stanislawski T Voss RH Lotz C Sadovnikova E Willemsen RA Kuball J. Circumventing tolerance to a human MDM2-derived tumor antigen by TCR gene transfer. Nat Immunol. (2001) 2:962–70. doi: 10.1038/ni1001-962, PMID: 11577350

[81] Johnson LA Morgan RA Dudley ME Cassard L Yang JC Hughes MS. Gene therapy with human and mouse T-cell receptors mediates cancer regression and targets normal tissues expressing cognate antigen. Blood. (2009) 114:535–46. doi: 10.1182/blood-2009-03-211714, PMID: 19451549 PMC2929689

[82] Hsiue EH Wright KM Douglass J Hwang MS Mog BJ Pearlman AH. Targeting a neoantigen derived from a common TP53 mutation. Science. (2021) 371:eabc8697. doi: 10.1126/science.abc8697, PMID: 33649166 PMC8208645

[83] Shen Y Li YM Zhou JJ Zhou Z Xu YC Zhao WB. The antitumor activity of TCR-mimic antibody-drug conjugates (TCRm-ADCs) targeting the intracellular wilms tumor 1 (WT1) oncoprotein. Int J Mol Sci. (2019) 20:3912. doi: 10.3390/ijms20163912, PMID: 31408937 PMC6720711

[84] Hoseini SS Cheung NK. Acute myeloid leukemia targets for bispecific antibodies. Blood Cancer J. (2017) 7:e552. doi: 10.1038/bcj.2017.35, PMID: 28409770 PMC5436078

[85] Bentzen AK Marquard AM Lyngaa R Saini SK Ramskov S Donia M. Large-scale detection of antigen-specific T cells using peptide-MHC-I multimers labeled with DNA barcodes. Nat Biotechnol. (2016) 34:1037–45. doi: 10.1038/nbt.3662, PMID: 27571370

[86] Bedran G Gasser HC Weke K Wang T Bedran D Laird A. The immunopeptidome from a genomic perspective: establishing the noncanonical landscape of MHC class I-associated peptides. Cancer Immunol Res. (2023) 11:747–62. doi: 10.1158/2326-6066.CIR-22-0621, PMID: 36961404 PMC10236148

[87] Gomase V S Sharma R Dhamane SP. Innovative immunoinformatics tools for enhancing MHC (Major histocompatibility complex) class I epitope prediction in immunoproteomics. Protein Pept Lett. (2025). doi: 10.2174/0109298665373152250625054723, PMID: 40662558

[88] Schmid DA Irving MB Posevitz V Hebeisen M Posevitz-Fejfar A Sarria JC. Evidence for a TCR affinity threshold delimiting maximal CD8 T cell function. J Immunol. (2010) 184:4936–46. doi: 10.4049/jimmunol.1000173, PMID: 20351194

[89] Stone JD Chervin AS Kranz DM. T-cell receptor binding affinities and kinetics: impact on T-cell activity and specificity. Immunology. (2009) 126:165–76. doi: 10.1111/j.1365-2567.2008.03015.x, PMID: 19125887 PMC2632691

[90] Li D Bentley C Yates J Salimi M Greig J Wiblin S. Engineering chimeric human and mouse major histocompatibility complex (MHC) class I tetramers for the production of T-cell receptor (TCR) mimic antibodies. PLoS One. (2017) 12:e0176642. doi: 10.1371/journal.pone.0176642, PMID: 28448627 PMC5407768

[91] Høydahl LS Berntzen G Løset GÅ. Engineering T-cell receptor-like antibodies for biologics and cell therapy. Curr Opin Biotechnol. (2024) 90:103224. doi: 10.1016/j.copbio.2024.103224, PMID: 39488859

[92] McCue AC Demarest SJ Froning KJ Hickey MJ Antonysamy S Kuhlman B. Engineering a tumor-selective prodrug T-cell engager bispecific antibody for safer immunotherapy. MAbs. (2024) 16:2373325. doi: 10.1080/19420862.2024.2373325, PMID: 38962811 PMC11225918

[93] Marcu A Bichmann L Kuchenbecker L Kowalewski DJ Freudenmann LK Backert L. HLA Ligand Atlas: a benign reference of HLA-presented peptides to improve T-cell-based cancer immunotherapy. J Immunother Cancer. (2021) 9:e002071. doi: 10.1136/jitc-2020-002071, PMID: 33858848 PMC8054196

[94] Ran Z Mu M Lin S Wang T Zeng J Kuang L. Deciphering the MHC immunopeptidome of human cancers with Ligand.MHC atlas. Brief Bioinform. (2025) 26:bbaf314. doi: 10.1093/bib/bbaf314, PMID: 40632497 PMC12239622

[95] Harper J Adams KJ Bossi G Wright DE Stacey AR Bedke N. An approved *in vitro* approach to preclinical safety and efficacy evaluation of engineered T cell receptor anti-CD3 bispecific (ImmTAC) molecules. PLoS One. (2018) 13:e0205491. doi: 10.1371/journal.pone.0205491, PMID: 30321203 PMC6188753

[96] Oates J Hassan NJ Jakobsen BK. ImmTACs for targeted cancer therapy: Why, what, how, and which. Mol Immunol. (2015) 67:67–74. doi: 10.1016/j.molimm.2015.01.024, PMID: 25708206

[97] Staeger R Tastanova A Ghosh A Winkelbeiner N Shukla P Kolm I. Tebentafusp elicits on-target cutaneous immune responses driven by cytotoxic T cells in uveal melanoma patients. J Clin Invest. (2025) 135:e181464. doi: 10.1172/JCI181464, PMID: 40311102 PMC12165791

[98] Ziogas DC Foteinou D Theocharopoulos C Martinos A Petsiou DP Anastasopoulou A. State-of-the-art in metastatic uveal melanoma treatment: A 2025 update: how to treat metastatic uveal melanoma in 2025. Curr Oncol Rep. (2025)27:803–21. doi: 10.1007/s11912-025-01684-0, PMID: 40380030 PMC12328531

[99] Vitek L Goronflot T Dutriaux C Deleuze A Le Corre Y Duval-Modeste AB. Efficacy and tolerability of tebentafusp in metastatic uveal melanoma: A real-life retrospective multicentre study. Acta Derm Venereol. (2024) 104:adv41297. doi: 10.2340/actadv.v104.41297, PMID: 39670438 PMC11681141

[100] Dao T Yan S Veomett N Pankov D Zhou L Korontsvit T. Targeting the intracellular WT1 oncogene product with a therapeutic human antibody. Sci Transl Med. (2013) 5:176ra33. doi: 10.1126/scitranslmed.3005661, PMID: 23486779 PMC3963696

[101] Liu X Xu Y Xiong W Yin B Huang Y Chu J. Development of a TCR-like antibody and chimeric antigen receptor against NY-ESO-1/HLA-A2 for cancer immunotherapy. J Immunother Cancer. (2022) 10:e004035. doi: 10.1136/jitc-2021-004035, PMID: 35338087 PMC8961179

[102] Saeed M van Brakel M Zalba S Schooten E Rens JA Koning GA. Targeting melanoma with immunoliposomes coupled to anti-MAGE A1 TCR-like single-chain antibody. Int J Nanomedicine. (2016) 11:955–75. doi: 10.2147/IJN.S96123, PMID: 27022262 PMC4792179

[103] Bassan D Gozlan YM Sharbi-Yunger A Tzehoval E Greenstein E Bitan L. Avidity optimization of a MAGE-A1-specific TCR with somatic hypermutation. Eur J Immunol. (2021) 51:1505–18. doi: 10.1002/eji.202049007, PMID: 33835499 PMC8252751

[104] Kirkey DC Loeb AM Castro S McKay CN Perkins L Pardo L. Therapeutic targeting of PRAME with mTCRCAR T cells in acute myeloid leukemia. Blood Adv. (2023) 7:1178–89. doi: 10.1182/bloodadvances.2022008304, PMID: 35984639 PMC10111362

[105] Naik A Thomas R Al-Khadairi G Bacha R Hendrickx W Decock J. Cancer testis antigen PRAME: An anti-cancer target with immunomodulatory potential. J Cell Mol Med. (2021) 25:10376–88. doi: 10.1111/jcmm.16967, PMID: 34612587 PMC8581324

[106] Lai J Wang Y Wu SS Ding D Sun ZY Zhang Y. Elimination of melanoma by sortase A-generated TCR-like antibody-drug conjugates (TL-ADCs) targeting intracellular melanoma antigen MART-1. Biomaterials. (2018) 178:158–69. doi: 10.1016/j.biomaterials.2018.06.017, PMID: 29933102

[107] Borbulevych OY Santhanagopolan SM Hossain M Baker BM. TCRs used in cancer gene therapy cross-react with MART-1/Melan-A tumor antigens via distinct mechanisms. J Immunol. (2011) 187:2453–63. doi: 10.4049/jimmunol.1101268, PMID: 21795600 PMC3166883

[108] Dao T Mun S Korontsvit T Khan AG Pohl MA White T. A TCR mimic monoclonal antibody for the HPV-16 E7-epitope p11-19/HLA-A*02:01 complex. PLoS One. (2022) 17:e0265534. doi: 10.1371/journal.pone.0265534, PMID: 35298559 PMC8929633

[109] Liu C Liu H Dasgupta M Hellman LM Zhang X Qu K. Validation and promise of a TCR mimic antibody for cancer immunotherapy of hepatocellular carcinoma. Sci Rep. (2022) 12:12068. doi: 10.1038/s41598-022-15946-5, PMID: 35840635 PMC9287321

[110] Zvyagin IV Tsvetkov VO Chudakov DM Shugay M. An overview of immunoinformatics approaches and databases linking T cell receptor repertoires to their antigen specificity. Immunogenetics. (2020) 72:77–84. doi: 10.1007/s00251-019-01139-4, PMID: 31741011

[111] Wu X Sereno AJ Huang F Zhang K Batt M Fitchett JR. Protein design of IgG/TCR chimeras for the co-expression of Fab-like moieties within bispecific antibodies. MAbs. (2015) 7:364–76. doi: 10.1080/19420862.2015.1007826, PMID: 25611120 PMC4623437

[112] Zhao WB Shen Y Liu WH Li YM Jin SJ Xu YC. Soluble expression of fc-fused T cell receptors allows yielding novel bispecific T cell engagers. Biomedicines. (2021) 9:790. doi: 10.3390/biomedicines9070790, PMID: 34356854 PMC8301436

[113] Malviya M Aretz ZEH Molvi Z Lee J Pierre S Wallisch P. Challenges and solutions for therapeutic TCR-based agents. Immunol Rev. (2023) 320:58–82. doi: 10.1111/imr.13233, PMID: 37455333 PMC11141734

[114] Shusta EV Holler PD Kieke MC Kranz DM Wittrup KD. Directed evolution of a stable scaffold for T-cell receptor engineering. Nat Biotechnol. (2000) 18:754–9. doi: 10.1038/77325, PMID: 10888844

[115] Raman MC Rizkallah PJ Simmons R Donnellan Z Dukes J Bossi G. Direct molecular mimicry enables off-target cardiovascular toxicity by an enhanced affinity TCR designed for cancer immunotherapy. Sci Rep. (2016) 6:18851. doi: 10.1038/srep18851, PMID: 26758806 PMC4725365

[116] Ratanji KD Derrick JP Dearman RJ Kimber I. Immunogenicity of therapeutic proteins: influence of aggregation. J Immunotoxicol. (2014) 11:99–109. doi: 10.3109/1547691X.2013.821564, PMID: 23919460 PMC4002659

[117] Thorn CR Bhattacharya D Crawford L Lin V Badkar A Kolhe P. Assessing the impact of viscosity lowering excipient on liquid-liquid phase separation for high concentration monoclonal antibody solutions. J Pharm Sci. (2025) 114:103804. doi: 10.1016/j.xphs.2025.103804, PMID: 40320242

[118] Ahmad A Refaat H Bhattacharya S Gurvich VJ Rathore AS Nejadnik R. Effect of formulation composition on trastuzumab stability. Int J Pharm. (2025) 671:125275. doi: 10.1016/j.ijpharm.2025.125275, PMID: 39870255

[119] Nathan P Hassel JC Rutkowski P Baurain JF Butler MO Schlaak M. Overall survival benefit with tebentafusp in metastatic uveal melanoma. N Engl J Med. (2021) 385:1196–206. doi: 10.1056/NEJMoa2103485, PMID: 34551229

[120] Wermke M Araujo DM Chatterjee M Tsimberidou AM Holderried TAW Jazaeri AA. Autologous T cell therapy for PRAME+ advanced solid tumors in HLA-A*02+ patients: a phase 1 trial. Nat Med. (2025) 31:2365–74. doi: 10.1038/s41591-025-03650-6, PMID: 40205198 PMC12283372

[121] Burt R Warcel D Fielding AK. Blinatumomab, a bispecific B-cell and T-cell engaging antibody, in the treatment of B-cell Malignancies. Hum Vaccin Immunother. (2019) 15:594–602. doi: 10.1080/21645515.2018.1540828, PMID: 30380973 PMC6605719

[122] Cameron BJ Gerry AB Dukes J Harper JV Kannan V Bianchi FC. Identification of a Titin-derived HLA-A1-presented peptide as a cross-reactive target for engineered MAGE A3-directed T cells. Sci Transl Med. (2013) 5:197ra103. doi: 10.1126/scitranslmed.3006034, PMID: 23926201 PMC6002776

[123] Householder KD Xiang X Jude KM Deng A Obenaus M Wilson SC. *De novo* design and structure of a peptide-centric TCR mimic binding module. bioRxiv [Preprint]. (2025) 14:2024. doi: 10.1101/2024.12.16.628822, PMID: 40705894 PMC12313176

[124] Ellington AD Szostak JW. *In vitro* selection of RNA molecules that bind specific ligands. Nature. (1990) 346:818–22. doi: 10.1038/346818a0, PMID: 1697402

[125] Zhou J Rossi J. Aptamers as targeted therapeutics: current potential and challenges. Nat Rev Drug Discov. (2017) 16:181–202. doi: 10.1038/nrd.2016.199, PMID: 27807347 PMC5700751

[126] Jungfleisch J Gebauer F. RNA-binding proteins as therapeutic targets in cancer. RNA Biol. (2025) 22:1–8. doi: 10.1080/15476286.2025.2470511, PMID: 40016176 PMC11869776

[127] Zhu G Ye M Donovan MJ Song E Zhao Z Tan W. Nucleic acid aptamers: an emerging frontier in cancer therapy. Chem Commun (Camb). (2012) 48:10472–80. doi: 10.1039/c2cc35042d, PMID: 22951893 PMC3869973

[128] Xiang D Shigdar S Qiao G Wang T Kouzani AZ Zhou SF. Nucleic acid aptamer-guided cancer therapeutics and diagnostics: the next generation of cancer medicine. Theranostics. (2015) 5:23–42. doi: 10.7150/thno.10202, PMID: 25553096 PMC4265746

[129] Aljohani MM Cialla-May D Popp J Chinnappan R Al-Kattan K Zourob M. Aptamers: potential diagnostic and therapeutic agents for blood diseases. Molecules. (2022) 27:383. doi: 10.3390/molecules27020383, PMID: 35056696 PMC8778139

[130] Chen Z Luo H Gubu A Yu S Zhang H Dai H. Chemically modified aptamers for improving binding affinity to the target proteins via enhanced non-covalent bonding. Front Cell Dev Biol. (2023) 11:1091809. doi: 10.3389/fcell.2023.1091809, PMID: 36910146 PMC9996316

[131] McNamara JO Kolonias D Pastor F Mittler RS Chen L Giangrande PH. Multivalent 4-1BB binding aptamers costimulate CD8+ T cells and inhibit tumor growth in mice. J Clin Invest. (2008) 118:376–86. doi: 10.1172/JCI33365, PMID: 18060045 PMC2104483

[132] Dollins CM Nair S Boczkowski D Lee J Layzer JM Gilboa E. Assembling OX40 aptamers on a molecular scaffold to create a receptor-activating aptamer. Chem Biol. (2008) 15:675–82. doi: 10.1016/j.chembiol.2008.05.016, PMID: 18635004 PMC2525512

[133] Passariello M Camorani S Vetrei C Cerchia L De Lorenzo C. Novel human bispecific aptamer-antibody conjugates for efficient cancer cell killing. Cancers (Basel). (2019) 11:1268. doi: 10.3390/cancers11091268, PMID: 31470510 PMC6770524

[134] Thomas BJ Porciani D Burke DH. Cancer immunomodulation using bispecific aptamers. Mol Ther Nucleic Acids. (2022) 27:894–915. doi: 10.1016/j.omtn.2022.01.008, PMID: 35141049 PMC8803965

[135] Boltz A Piater B Toleikis L Guenther R Kolmar H Hock B. Bi-specific aptamers mediating tumor cell lysis. J Biol Chem. (2011) 286:21896–905. doi: 10.1074/jbc.M111.238261, PMID: 21531729 PMC3122244

[136] Schrand B Berezhnoy A Brenneman R Williams A Levay A Kong LY. Targeting 4-1BB costimulation to the tumor stroma with bispecific aptamer conjugates enhances the therapeutic index of tumor immunotherapy. Cancer Immunol Res. (2014) 2:867–77. doi: 10.1158/2326-6066.CIR-14-0007, PMID: 24938283 PMC5502823

[137] Cartwright AN Griggs J Davis DM. The immune synapse clears and excludes molecules above a size threshold. Nat Commun. (2014) 5:5479. doi: 10.1038/ncomms6479, PMID: 25407222 PMC4248232

[138] Dickopf S Georges GJ Brinkmann U. Format and geometries matter: Structure-based design defines the functionality of bispecific antibodies. Comput Struct Biotechnol J. (2020) 18:1221–7. doi: 10.1016/j.csbj.2020.05.006, PMID: 32542108 PMC7283971

[139] Vinay DS Ryan EP Pawelec G Talib WH Stagg J Elkord E. Immune evasion in cancer: Mechanistic basis and therapeutic strategies. Semin Cancer Biol. (2015) 35 Suppl:S185–98. doi: 10.1016/j.semcancer.2015.03.004, PMID: 25818339

[140] Pastor F Soldevilla MM Villanueva H Kolonias D Inoges S de Cerio AL. CD28 aptamers as powerful immune response modulators. Mol Ther Nucleic Acids. (2013) 2:e98. doi: 10.1038/mtna.2013.26, PMID: 23756353 PMC3696906

[141] Pratico ED Sullenger BA Nair SK. Identification and characterization of an agonistic aptamer against the T cell costimulatory receptor, OX40. Nucleic Acid Ther. (2013) 23:35–43. doi: 10.1089/nat.2012.0388, PMID: 23113766 PMC3569954

[142] Hoffmann MM Molina-Mendiola C Nelson AD Parks CA Reyes EE Hansen MJ. Co-potentiation of antigen recognition: A mechanism to boost weak T cell responses and provide immunotherapy *in vivo* . Sci Adv. (2015) 1:e1500415. doi: 10.1126/sciadv.1500415, PMID: 26601285 PMC4646799

[143] Yeap WH Wong KL Shimasaki N Teo EC Quek JK Yong HX. CD16 is indispensable for antibody-dependent cellular cytotoxicity by human monocytes. Sci Rep. (2016) 6:34310. doi: 10.1038/srep34310, PMID: 27670158 PMC5037471

[144] Li Z Hu Y An Y Duan J Li X Yang XD. Novel bispecific aptamer enhances immune cytotoxicity against MUC1-positive tumor cells by MUC1-CD16 dual targeting. Molecules. (2019) 24:478. doi: 10.3390/molecules24030478, PMID: 30699986 PMC6385031

[145] Prodeus A Abdul-Wahid A Fischer NW Huang EH Cydzik M Gariépy J. Targeting the PD-1/PD-L1 immune evasion axis with DNA aptamers as a novel therapeutic strategy for the treatment of disseminated cancers. Mol Ther Nucleic Acids. (2015) 4:e237. doi: 10.1038/mtna.2015.11, PMID: 25919090 PMC4417124

[146] Li ZF Chen C Zeng JY Wang S Han SQ Zhang YQ. Screening and characterization of aptamers for recombinant human programmed death-1 and recombinant extracellular domain of human programmed death ligand-1. Eur Rev Med Pharmacol Sci. (2021) 25:3997–4004. doi: 10.26355/eurrev_202106_26040, PMID: 34156677

[147] Huang BT Lai WY Chang YC Wang JW Yeh SD Lin EP. A CTLA-4 antagonizing DNA aptamer with antitumor effect. Mol Ther Nucleic Acids. (2017) 8:520–8. doi: 10.1016/j.omtn.2017.08.006, PMID: 28918052 PMC5573796

[148] Gefen T Castro I Muharemagic D Puplampu-Dove Y Patel S Gilboa E. A TIM-3 oligonucleotide aptamer enhances T cell functions and potentiates tumor immunity in mice. Mol Ther. (2017) 25:2280–8. doi: 10.1016/j.ymthe.2017.06.023, PMID: 28800954 PMC5628791

[149] Ajona D Ortiz-Espinosa S Moreno H Lozano T Pajares MJ Agorreta J. A combined PD-1/C5a blockade synergistically protects against lung cancer growth and metastasis. Cancer Discov. (2017) 7:694–703. doi: 10.1158/2159-8290.CD-16-1184, PMID: 28288993

[150] Miao Y Gao Q Mao M Zhang C Yang L Yang Y. Bispecific aptamer chimeras enable targeted protein degradation on cell membranes. Angew Chem Int Ed Engl. (2021) 60:11267–71. doi: 10.1002/anie.202102170, PMID: 33634555

[151] Wang L Liang H Sun J Liu Y Li J Li J. Bispecific aptamer induced artificial protein-pairing: A strategy for selective inhibition of receptor function. J Am Chem Soc. (2019) 141:12673–81. doi: 10.1021/jacs.9b05123, PMID: 31381313

[152] Soldevilla MM Villanueva H Casares N Lasarte JJ Bendandi M Inoges S. MRP1-CD28 bi-specific oligonucleotide aptamers: target costimulation to drug-resistant melanoma cancer stem cells. Oncotarget. (2016) 7:23182–96. doi: 10.18632/oncotarget.8095, PMID: 26992239 PMC5029619

[153] Vater A Klussmann S. Turning mirror-image oligonucleotides into drugs: the evolution of Spiegelmer(^®^) therapeutics. Drug Discov Today. (2015) 20:147–55. doi: 10.1016/j.drudis.2014.09.004, PMID: 25236655

[154] Ayass MA Tripathi T Griko N Okyay T Ramankutty Nair R Zhang J. Dual checkpoint aptamer immunotherapy: unveiling tailored cancer treatment targeting CTLA-4 and NKG2A. Cancers (Basel). (2024) 16:1041. doi: 10.3390/cancers16051041, PMID: 38473398 PMC10931446

[155] Choi SI Lee YS Lee YM Kim HJ Kim WJ Jung S. Complexation of drug and hapten-conjugated aptamer with universal hapten antibody for pancreatic cancer treatment. J Control Release. (2023) 360:940–52. doi: 10.1016/j.jconrel.2023.03.048, PMID: 37001565

[156] Rosenberg JE Bambury RM Van Allen EM Drabkin HA Lara PN Jr Harzstark AL. A phase II trial of AS1411 (a novel nucleolin-targeted DNA aptamer) in metastatic renal cell carcinoma. Invest New Drugs. (2014) 32:178–87. doi: 10.1007/s10637-013-0045-6, PMID: 24242861 PMC4560460

[157] Giordano FA Layer JP Leonardelli S Friker LL Turiello R Corvino D. L-RNA aptamer-based CXCL12 inhibition combined with radiotherapy in newly-diagnosed glioblastoma: dose escalation of the phase I/II GLORIA trial. Nat Commun. (2024) 15:4210. doi: 10.1038/s41467-024-48416-9, PMID: 38806504 PMC11133480

[158] Elskens JP Elskens JM Madder A. Chemical modification of aptamers for increased binding affinity in diagnostic applications: current status and future prospects. Int J Mol Sci. (2020) 21:4522. doi: 10.3390/ijms21124522, PMID: 32630547 PMC7350236

[159] Kovacevic KD Gilbert JC Jilma B. Pharmacokinetics, pharmacodynamics and safety of aptamers. Adv Drug Delivery Rev. (2018) 134:36–50. doi: 10.1016/j.addr.2018.10.008, PMID: 30321620

[160] Harding FA Stickler MM Razo J DuBridge RB. The immunogenicity of humanized and fully human antibodies: residual immunogenicity resides in the CDR regions. MAbs. (2010) 2:256–65. doi: 10.4161/mabs.2.3.11641, PMID: 20400861 PMC2881252

[161] Wang Z Yang X Lee NZ Cao X. Multivalent aptamer approach: designs, strategies, and applications. Micromachines (Basel). (2022) 13:436. doi: 10.3390/mi13030436, PMID: 35334728 PMC8956053

[162] Moradi Z Abnous K Taghdisi SM Zamanian J Moshiri M Etemad D. Designing multivalent aptamers: Recent advancements in diagnostic and therapeutic approaches for cancer treatment. J Drug Delivery Sci Technol. (2025) 105:106614. doi: 10.1016/j.jddst.2025.106614

[163] Zhang Y Lai BS Juhas M. Recent advances in aptamer discovery and applications. Molecules. (2019) 24:941. doi: 10.3390/molecules24050941, PMID: 30866536 PMC6429292

[164] Rajasekaran SA Anilkumar G Oshima E Bowie JU Liu H Heston W. A novel cytoplasmic tail MXXXL motif mediates the internalization of prostate-specific membrane antigen. Mol Biol Cell. (2003) 14:4835–45. doi: 10.1091/mbc.e02-11-0731, PMID: 14528023 PMC284788

[165] Liu S Li X Gao H Chen J Jiang H. Progress in aptamer research and future applications. ChemistryOpen. (2025) 14:e202400463. doi: 10.1002/open.202400463, PMID: 39901496 PMC13140418

[166] Zhao Y Chen G Li X Wu J Chang B Hu S. KN046, a bispecific antibody against PD-L1 and CTLA-4, plus chemotherapy as first-line treatment for metastatic NSCLC: A multicenter phase 2 trial. Cell Rep Med. (2024) 5:101470. doi: 10.1016/j.xcrm.2024.101470, PMID: 38508135 PMC10983105

[167] Mojarad-Jabali S Farshbaf M Walker PR Hemmati S Fatahi Y Zakeri-Milani P. An update on actively targeted liposomes in advanced drug delivery to glioma. Int J Pharm. (2021) 602:120645. doi: 10.1016/j.ijpharm.2021.120645, PMID: 33915182

[168] Xie S Wang Z Fu T Zheng L Wu H He L. Engineering aptamers with selectively enhanced biostability in the tumor microenvironment. Angew Chem Int Ed Engl. (2022) 61:e202201220. doi: 10.1002/anie.202201220, PMID: 35536294

[169] Qin S Xu L Yi M Yu S Wu K Luo S. Novel immune checkpoint targets: moving beyond PD-1 and CTLA-4. Mol Cancer. (2019) 18:155. doi: 10.1186/s12943-019-1091-2, PMID: 31690319 PMC6833286

[170] Zheng J Zhao S Yu X Huang S Liu HY. Simultaneous targeting of CD44 and EpCAM with a bispecific aptamer effectively inhibits intraperitoneal ovarian cancer growth. Theranostics. (2017) 7:1373–88. doi: 10.7150/thno.17826, PMID: 28435472 PMC5399600

[171] Chen G Mao D Wang X Chen J Gu C Huang S. Aptamer-based self-assembled nanomicelle enables efficient and targeted drug delivery. J Nanobiotechnology. (2023) 21:415. doi: 10.1186/s12951-023-02164-y, PMID: 37946192 PMC10634091

[172] Zhang Y Dong Q Xiao J Fang X Huang W Li Q. In-vivo polyvalent aptamer@protein-based nanocarrier with synergistic charge effect for high drug loading, high nuclease resistance, and high receptor accessibility. CCS Chem. (2024)7:1411–23. doi: 10.31635/ccschem.023.202303640

[173] Chen Z Hu L Zhang BT Lu A Wang Y Yu Y. Artificial intelligence in aptamer-target binding prediction. Int J Mol Sci. (2021) 22:3605. doi: 10.3390/ijms22073605, PMID: 33808496 PMC8038094

[174] Zhao Z Xiao X Saw PE Wu W Huang H Chen J. Chimeric antigen receptor T cells in solid tumors: a war against the tumor microenvironment. Sci China Life Sci. (2020) 63:180–205. doi: 10.1007/s11427-019-9665-8, PMID: 31883066

[175] Slaney CY Wang P Darcy PK Kershaw MH. CARs versus biTEs: A comparison between T cell-redirection strategies for cancer treatment. Cancer Discov. (2018) 8:924–34. doi: 10.1158/2159-8290.CD-18-0297, PMID: 30012854

[176] Tabernero J Melero I Ros W Argiles G Marabelle A Rodriguez-Ruiz ME. Phase Ia and Ib studies of the novel carcinoembryonic antigen (CEA) T-cell bispecific (CEA CD3 TCB) antibody as a single agent and in combination with atezolizumab: Preliminary efficacy and safety in patients with metastatic colorectal cancer (mCRC). J Clin Oncol. (2017) 35:3002. doi: 10.1200/JCO.2017.35.15_suppl.3002 28644773

[177] Klinger M Brandl C Zugmaier G Hijazi Y Bargou RC Topp MS. Immunopharmacologic response of patients with B-lineage acute lymphoblastic leukemia to continuous infusion of T cell-engaging CD19/CD3-bispecific BiTE antibody blinatumomab. Blood. (2012) 119:6226–33. doi: 10.1182/blood-2012-01-40051, PMID: 22592608

[178] Kebenko M Goebeler ME Wolf M Hasenburg A Seggewiss-Bernhardt R Ritter B. A multicenter phase 1 study of solitomab (MT110, AMG 110), a bispecific EpCAM/CD3 T-cell engager (BiTE^®^) antibody construct, in patients with refractory solid tumors. Oncoimmunology. (2018) 7:e1450710. doi: 10.1080/2162402X.2018.1450710, PMID: 30221040 PMC6136859

[179] Pishvaian M Morse MA McDevitt J Norton JD Ren S Robbie GJ. Phase 1 dose escalation study of MEDI-565, a bispecific T-cell engager that targets human carcinoembryonic antigen, in patients with advanced gastrointestinal adenocarcinomas. Clin Colorectal Cancer. (2016) 15:345–51. doi: 10.1016/j.clcc.2016.07.009, PMID: 27591895

[180] Middleton MR McAlpine C Woodcock VK Corrie P Infante JR Steven NM. Tebentafusp, A TCR/anti-CD3 bispecific fusion protein targeting gp100, potently activated antitumor immune responses in patients with metastatic melanoma. Clin Cancer Res. (2020) 26:5869–78. doi: 10.1158/1078-0432.CCR-20-1247, PMID: 32816891 PMC9210997

[181] Basse C Chabanol H Bonte PE Fromantin I Girard N. Management of cutaneous toxicities under amivantamab (anti MET and anti EGFR bispecific antibody) in patients with metastatic non-small cell lung cancer harboring EGFR Exon20ins: towards a proactive, multidisciplinary approach. Lung Cancer. (2022) 173:116–23. doi: 10.1016/j.lungcan.2022.09.012, PMID: 36198244

[182] Bluemel C Hausmann S Fluhr P Sriskandarajah M Stallcup WB Baeuerle PA. Epitope distance to the target cell membrane and antigen size determine the potency of T cell-mediated lysis by BiTE antibodies specific for a large melanoma surface antigen. Cancer Immunol Immunother. (2010) 59:1197–209. doi: 10.1007/s00262-010-0844-y, PMID: 20309546 PMC11030089

[183] Panchal A Seto P Wall R Hillier BJ Zhu Y Krakow J. COBRA™: a highly potent conditionally active T cell engager engineered for the treatment of solid tumors. MAbs. (2020) 12:1792130. doi: 10.1080/19420862.2020.1792130, PMID: 32684124 PMC7531513

[184] Cattaruzza F Nazeer A To M Hammond M Koski C Liu LY. Precision-activated T-cell engagers targeting HER2 or EGFR and CD3 mitigate on-target, off-tumor toxicity for immunotherapy in solid tumors. Nat Cancer. (2023) 4:485–501. doi: 10.1038/s43018-023-00536-9, PMID: 36997747 PMC10132983

[185] Phillips TJ Carlo-Stella C Morschhauser F Bachy E Crump M Trněný M. Glofitamab in relapsed/refractory mantle cell lymphoma: results from a phase I/II study. J Clin Oncol. (2025) 43:318–28. doi: 10.1200/JCO.23.02470, PMID: 39365960 PMC11771347

[186] Cheema M Syed S Ghuman Z Iftikhar A. Teclistamab-associated cytokine release syndrome in multiple myeloma: a case-based literature review of mechanisms, management, and clinical implications. Arch Clin Cases. (2025) 12:98–101. doi: 10.22551/2025.47.1202.10320, PMID: 40666817 PMC12262052

[187] Parkhurst MR Yang JC Langan RC Dudley ME Nathan DA Feldman SA. T cells targeting carcinoembryonic antigen can mediate regression of metastatic colorectal cancer but induce severe transient colitis. Mol Ther. (2011) 19:620–6. doi: 10.1038/mt.2010.272, PMID: 21157437 PMC3048186

[188] Lee CH Salio M Napolitani G Ogg G Simmons A Koohy H. Predicting cross-reactivity and antigen specificity of T cell receptors. Front Immunol. (2020) 11:565096. doi: 10.3389/fimmu.2020.565096, PMID: 33193332 PMC7642207

[189] Antunes DA Baker BM Cornberg M Selin LK. Editorial: Quantification and prediction of T-cell cross-reactivity through experimental and computational methods. Front Immunol. (2024) 15:1377259. doi: 10.3389/fimmu.2024.1377259, PMID: 38444853 PMC10912571

[190] Stone JD Harris DT Kranz DM. TCR affinity for p/MHC formed by tumor antigens that are self-proteins: impact on efficacy and toxicity. Curr Opin Immunol. (2015) 3:16–22. doi: 10.1016/j.coi.2015.01.003, PMID: 25618219 PMC4920053

[191] Spear TT Evavold BD Baker BM Nishimura MI. Understanding TCR affinity, antigen specificity, and cross-reactivity to improve TCR gene-modified T cells for cancer immunotherapy. Cancer Immunol Immunother. (2019) 68:1881–9. doi: 10.1007/s00262-019-02401-0, PMID: 31595324 PMC11028285

[192] Gust J Hay KA Hanafi LA Li D Myerson D Gonzalez-Cuyar LF. Endothelial activation and blood-brain barrier disruption in neurotoxicity after adoptive immunotherapy with CD19 CAR-T cells. Cancer Discov. (2017) 7:1404–19. doi: 10.1158/2159-8290.CD-17-0698, PMID: 29025771 PMC5718945

[193] Santomasso BD Park JH Salloum D Riviere I Flynn J Mead E. Clinical and biological correlates of neurotoxicity associated with CAR T-cell therapy in patients with B-cell acute lymphoblastic leukemia. Cancer Discov. (2018) 8:958–71. doi: 10.1158/2159-8290.CD-17-1319, PMID: 29880584 PMC6385599

[194] Jain RK. Normalizing tumor microenvironment to treat cancer: bench to bedside to biomarkers. J Clin Oncol. (2013) 31:2205–18. doi: 10.1200/JCO.2012.46.3653, PMID: 23669226 PMC3731977

[195] Thurber GM Schmidt MM Wittrup KD. Antibody tumor penetration: transport opposed by systemic and antigen-mediated clearance. Adv Drug Delivery Rev. (2008) 60:1421–34. doi: 10.1016/j.addr.2008.04.012, PMID: 18541331 PMC2820307

[196] Shukla AA Norman C. Downstream processing of Fc fusion proteins, bispecific antibodies, and antibody–drug conjugates. Gottschalk U, editor. Hoboken, New Jersey: Wiley. (2017). doi: 10.1002/9781119126942.ch26

[197] Heldin CH Rubin K Pietras K Ostman A. High interstitial fluid pressure - an obstacle in cancer therapy. Nat Rev Cancer. (2004) 4:806–13. doi: 10.1038/nrc1456, PMID: 15510161

[198] Yi M Wu Y Niu M Zhu S Zhang J Yan Y. Anti-TGF-β/PD-L1 bispecific antibody promotes T cell infiltration and exhibits enhanced antitumor activity in triple-negative breast cancer. J Immunother Cancer. (2022) 10:e005543. doi: 10.1136/jitc-2022-005543, PMID: 36460337 PMC9723957

[199] Park JA Espinosa-Cotton M Guo HF Monette S Cheung NV. Targeting tumor vasculature to improve antitumor activity of T cells armed ex vivo with T cell engaging bispecific antibody. J Immunother Cancer. (2023) 11:e006680. doi: 10.1136/jitc-2023-006680, PMID: 36990507 PMC10069597

[200] Banks WA. From blood-brain barrier to blood-brain interface: new opportunities for CNS drug delivery. Nat Rev Drug Discov. (2016) 15:275–92. doi: 10.1038/nrd.2015.21, PMID: 26794270

[201] Sternjak A Lee F Thomas O Balazs M Wahl J Lorenczewski G. Preclinical assessment of AMG 596, a bispecific T-cell engager (BiTE) immunotherapy targeting the tumor-specific antigen EGFRvIII. Mol Cancer Ther. (2021) 20:925–33. doi: 10.1158/1535-7163.MCT-20-0508, PMID: 33632870

[202] Kontermann RE. Strategies for extended serum half-life of protein therapeutics. Curr Opin Biotechnol. (2011) 22:868–76. doi: 10.1016/j.copbio.2011.06.012, PMID: 21862310

[203] Provenzano PP Cuevas C Chang AE Goel VK Von Hoff DD Hingorani SR. Enzymatic targeting of the stroma ablates physical barriers to treatment of pancreatic ductal adenocarcinoma. Cancer Cell. (2012) 21:418–29. doi: 10.1016/j.ccr.2012.01.007, PMID: 22439937 PMC3371414

[204] Desnoyers LR Vasiljeva O Richardson JH Yang A Menendez EE Liang TW. Tumor-specific activation of an EGFR-targeting probody enhances therapeutic index. Sci Transl Med. (2013) 5:207ra144. doi: 10.1126/scitranslmed.3006682, PMID: 24132639

[205] Bai XF Liu J Li O Zheng P Liu Y. Antigenic drift as a mechanism for tumor evasion of destruction by cytolytic T lymphocytes. J Clin Invest. (2003) 111:1487–96. doi: 10.1172/JCI17656, PMID: 12750398 PMC155049

[206] Gebhart G Lamberts LE Wimana Z Garcia C Emonts P Ameye L. Molecular imaging as a tool to investigate heterogeneity of advanced HER2-positive breast cancer and to predict patient outcome under trastuzumab emtansine (T-DM1): the ZEPHIR trial. Ann Oncol. (2016) 27:619–24. doi: 10.1093/annonc/mdv577, PMID: 26598545

[207] Dijkers EC Oude Munnink TH Kosterink JG Brouwers AH Jager PL de Jong JR. Biodistribution of 89Zr-trastuzumab and PET imaging of HER2-positive lesions in patients with metastatic breast cancer. Clin Pharmacol Ther. (2010) 87:586–92. doi: 10.1038/clpt.2010.12, PMID: 20357763

[208] Goebeler ME Bargou R. Blinatumomab: a CD19/CD3 bispecific T cell engager (BiTE) with unique anti-tumor efficacy. Leuk Lymphoma. (2016) 57:1021–32. doi: 10.3109/10428194.2016.1161185, PMID: 27050240

[209] Friedrich M Henn A Raum T Bajtus M Matthes K Hendrich L. Preclinical characterization of AMG 330, a CD3/CD33-bispecific T-cell-engaging antibody with potential for treatment of acute myelogenous leukemia. Mol Cancer Ther. (2014) 13:1549–57. doi: 10.1158/1535-7163.MCT-13-0956, PMID: 24674885

[210] Gerlinger M Rowan AJ Horswell S Math M Larkin J Endesfelder D. Intratumor heterogeneity and branched evolution revealed by multiregion sequencing. N Engl J Med. (2012) 366:883–92. doi: 10.1056/NEJMoa1113205, PMID: 22397650 PMC4878653

[211] Sabari JK Lok BH Laird JH Poirier JT Rudin CM. Unravelling the biology of SCLC: implications for therapy. Nat Rev Clin Oncol. (2017) 14:549–61. doi: 10.1038/nrclinonc.2017.71, PMID: 28534531 PMC5843484

[212] Janjigian YY Maron SB Chatila WK Millang B Chavan SS Alterman C. First-line pembrolizumab and trastuzumab in HER2-positive oesophageal, gastric, or gastro-oesophageal junction cancer: an open-label, single-arm, phase 2 trial. Lancet Oncol. (2020) 21:821–31. doi: 10.1016/S1470-2045(20)30169-8, PMID: 32437664 PMC8229851

[213] Yarmarkovich M Marshall QF Warrington JM Premaratne R Farrel A Groff D. Cross-HLA targeting of intracellular oncoproteins with peptide-centric CARs. Nature. (2021) 599:477–84. doi: 10.1038/s41586-021-04061-6, PMID: 34732890 PMC8599005

[214] Park GH Park S Kim H Jung HA Sun JM Ahn JS. Prospective investigation of biomarker and resistance mechanism using longitudinal cell-free NGS in non-small cell lung cancer with EGFR exon 20 insertion treated with amivantamab. Eur J Cancer. (2025) 226:115631. doi: 10.1016/j.ejca.2025.115631, PMID: 40651388

[215] Liu P Ding H Jia S Pang Y Li C Zhao T. Molecular imaging of B7-H3-targeting bispecific T cell-engaging antibody MGD009 in glioblastoma models. ACS Appl Mater Interfaces. (2025) 17:22384–93. doi: 10.1021/acsami.5c01451, PMID: 40183579

[216] Mega A Mebrahtu A Aniander G Ryer E Sköld A Sandegren A. A PDGFRB- and CD40-targeting bispecific AffiMab induces stroma-targeted immune cell activation. MAbs. (2023) 15:2223750. doi: 10.1080/19420862.2023.2223750, PMID: 37332119 PMC10332328

[217] Krishna M Nadler SG. Immunogenicity to biotherapeutics - the role of anti-drug immune complexes. Front Immunol. (2016) 7:21. doi: 10.3389/fimmu.2016.00021, PMID: 26870037 PMC4735944

[218] Davda J Declerck P Hu-Lieskovan S Hickling TP Jacobs IA Chou J. Immunogenicity of immunomodulatory, antibody-based, oncology therapeutics. J Immunother Cancer. (2019) 7:105. doi: 10.1186/s40425-019-0586-0, PMID: 30992085 PMC6466770

[219] Franquiz MJ Short NJ. Blinatumomab for the treatment of adult B-cell acute lymphoblastic leukemia: toward a new era of targeted immunotherapy. Biologics. (2020) 14:23–34. doi: 10.2147/BTT.S202746, PMID: 32103893 PMC7027838

[220] Lim K Zhu XS Zhou D Ren S Phipps A. Clinical pharmacology strategies for bispecific antibody development: learnings from FDA-approved bispecific antibodies in oncology. Clin Pharmacol Ther. (2024) 116:315–27. doi: 10.1002/cpt.3308, PMID: 38825990

[221] Piha-Paul S Bendell J Tolcher A Hurvitz S Patnaik A Shroff R. O82 A phase 1 dose escalation study of PRS-343, a HER2/4–1BB bispecific molecule, in patients with HER2-positive Malignancies. J Immunother. Cancer. (2020) 8:A1–2. doi: 10.1136/LBA2019.2

[222] Jurichson J. Aptevo Therapeutics and MorphoSys End Joint Development and Commercialization Agreement for MOR209/ES414. Seattle, WA, USA: Aptevo Therapeutics Inc (2017).

[223] Wu J Fu J Zhang M Liu D. AFM13: a first-in-class tetravalent bispecific anti-CD30/CD16A antibody for NK cell-mediated immunotherapy. J Hematol Oncol. (2015) 8:96. doi: 10.1186/s13045-015-0188-3, PMID: 26231785 PMC4522136

[224] Sotillo E Barrett DM Black KL Bagashev A Oldridge D Wu G. Convergence of acquired mutations and alternative splicing of CD19 enables resistance to CART-19 immunotherapy. Cancer Discov. (2015) 5:1282–95. doi: 10.1158/2159-8290.CD-15-1020, PMID: 26516065 PMC4670800

[225] Zhao L Li S Wei X Qi X Liu D Liu L. A novel CD19/CD22/CD3 trispecific antibody enhances therapeutic efficacy and overcomes immune escape against B-ALL. Blood. (2022) 140:1790–802. doi: 10.1182/blood.2022016243, PMID: 35981465

[226] Roddie C Lekakis LJ Marzolini MAV Ramakrishnan A Zhang Y Hu Y. Dual targeting of CD19 and CD22 with bicistronic CAR-T cells in patients with relapsed/refractory large B-cell lymphoma. Blood. (2023) 141:2470–82. doi: 10.1182/blood.2022018598, PMID: 36821767 PMC10646794

[227] Marschollek P Liszka K Mielcarek-Siedziuk M Rybka B Ryczan-Krawczyk R Panasiuk A. Blinatumomab prior to CAR-T cell therapy-A treatment option worth consideration for high disease burden. Biomedicines. (2022) 10:2915. doi: 10.3390/biomedicines10112915, PMID: 36428483 PMC9687755

[228] Glatte B Wenk K Grahnert A Friedrich M Merz M Vucinic V. Teclistamab impairs detection of BCMA CAR-T cells. Blood Adv. (2023) 7:3842–5. doi: 10.1182/bloodadvances.2023009714, PMID: 37026812 PMC10393749

[229] Sarkar S Rav E Stitzlein L Gibson A McCall D Nunez C. Palbociclib and chemotherapy followed by blinatumomab consolidation to CAR-T cell therapy in KMT2A-rearranged, therapy-related acute lymphoblastic leukemia. Pediatr Blood Cancer. (2024) 71:e30964. doi: 10.1002/pbc.30964, PMID: 38514796

[230] Merz M Dima D Hashmi H Ahmed N Stölzel F Holderried TAW. Bispecific antibodies targeting BCMA or GPRC5D are highly effective in relapsed myeloma after CAR T-cell therapy. Blood Cancer J. (2024) 14:214. doi: 10.1038/s41408-024-01197-2, PMID: 39632797 PMC11618392

[231] Güç E Treveil A Leach E Broomfield A Camera A Clubley J. Tebentafusp, a T cell engager, promotes macrophage reprogramming and in combination with IL-2 overcomes macrophage immunosuppression in cancer. Nat Commun. (2025) 16:2374. doi: 10.1038/s41467-025-57470-w, PMID: 40064880 PMC11893752

[232] Kerbauy LN Marin ND Kaplan M Banerjee PP Berrien-Elliott MM Becker-Hapak M. Combining AFM13, a bispecific CD30/CD16 antibody, with cytokine-activated blood and cord blood-derived NK cells facilitates CAR-like responses against CD30^+^ Malignancies. Clin Cancer Res. (2021) 27:3744–56. doi: 10.1158/1078-0432.CCR-21-0164, PMID: 33986022 PMC8254785

[233] Nieto Y Banerjee P Kaur I Basar R Li Y Daher M. Allogeneic NK cells with a bispecific innate cell engager in refractory relapsed lymphoma: a phase 1 trial. Nat Med. (2025) 31:1987–93. doi: 10.1038/s41591-025-03640-8, PMID: 40186077 PMC13218020

[234] Schinke C Touzeau C Minnema M van de Donk N Rodríguez-Otero P Mateos M. Pivotal phase 2 MonumenTAL-1 results of talquetamab (tal), a GPRC5DxCD3 bispecific antibody (BsAb), for relapsed/refractory multiple myeloma (RRMM). J Clin Oncol. (2023)41:8036. doi: 10.1200/JCO.2023.41.16_suppl.8036

[235] Garfall AL Dancy EK Cohen AD Hwang WT Fraietta JA Davis MM. T-cell phenotypes associated with effective CAR T-cell therapy in postinduction vs relapsed multiple myeloma. Blood Adv. (2019) 3:2812–5. doi: 10.1182/bloodadvances.2019000600, PMID: 31575532 PMC6784521

[236] Danhof S Schreder M Knop S Rasche L Strifler S Löffler C. Expression of programmed death-1 on lymphocytes in myeloma patients is lowered during lenalidomide maintenance. Haematologica. (2018) 103:e126–9. doi: 10.3324/haematol.2017.178947, PMID: 29191843 PMC5830376

[237] Müller D. Optimized CD19/CD22/CD3 antibody. Blood. (2022) 140:1750–1. doi: 10.1182/blood.2022018081, PMID: 36264591

[238] Pan D Kumar A Lipof JJ Chung A Wolf JL Martin TG 3rd. Emerging GPRC5D-Targeted therapies for multiple myeloma: a comprehensive review. Expert Opin Investig Drugs. (2025) 34:379–89. doi: 10.1080/13543784.2025.2511179, PMID: 40425184

[239] Devasia AJ Chari A Lancman G. Bispecific antibodies in the treatment of multiple myeloma. Blood Cancer J. (2024) 14:158. doi: 10.1038/s41408-024-01139-y, PMID: 39266530 PMC11393350

[240] Chang CH Wang Y Li R Rossi DL Liu D Rossi EA. Combination therapy with bispecific antibodies and PD-1 blockade enhances the antitumor potency of T cells. Cancer Res. (2017) 77:5384–94. doi: 10.1158/0008-5472.CAN-16-3431, PMID: 28819027

[241] Sam J Colombetti S Fauti T Roller A Biehl M Fahrni L. Combination of T-cell bispecific antibodies with PD-L1 checkpoint inhibition elicits superior anti-tumor activity. Front Oncol. (2020) 10:575737. doi: 10.3389/fonc.2020.575737, PMID: 33330050 PMC7735156

[242] Ribas A Wolchok JD. Cancer immunotherapy using checkpoint blockade. Science. (2018) 359:1350–5. doi: 10.1126/science.aar4060, PMID: 29567705 PMC7391259

[243] Hegde PS Chen DS. Top 10 challenges in cancer immunotherapy. Immunity. (2020) 52:17–35. doi: 10.1016/j.immuni.2019.12.011, PMID: 31940268

[244] Feucht J Kayser S Gorodezki D Hamieh M Döring M Blaeschke F. T-cell responses against CD19+ pediatric acute lymphoblastic leukemia mediated by bispecific T-cell engager (BiTE) are regulated contrarily by PD-L1 and CD80/CD86 on leukemic blasts. Oncotarget. (2016) 7:76902–19. doi: 10.18632/oncotarget.12357, PMID: 27708227 PMC5363558

[245] Lim SM Kang SS Kim DK Lee SH Synn CB Baek S. Exploration of immune-modulatory effects of amivantamab in combination with pembrolizumab in lung and head and neck squamous cell carcinoma. Cancer Res Commun. (2024) 4:1748–64. doi: 10.1158/2767-9764.CRC-24-0107, PMID: 38916448 PMC11253790

[246] Rubio-Pérez L Frago S Compte M Navarro R Harwood SL Lázaro-Gorines R. Characterization of a trispecific PD-L1 blocking antibody that exhibits EGFR-conditional 4-1BB agonist activity. Antibodies (Basel). (2024) 13:34. doi: 10.3390/antib13020034, PMID: 38804302 PMC11130918

[247] Yuwen H Wang H Li T Ren Y Zhang YK Chen P. ATG-101 is a tetravalent PD-L1×4-1BB bispecific antibody that stimulates antitumor immunity through PD-L1 blockade and PD-L1-directed 4-1BB activation. Cancer Res. (2024) 84:1680–98. doi: 10.1158/0008-5472.CAN-23-2701, PMID: 38501978 PMC11094422

[248] Peper-Gabriel JK Pavlidou M Pattarini L Morales-Kastresana A Jaquin TJ Gallou C. The PD-L1/4-1BB bispecific antibody-anticalin fusion protein PRS-344/S095012 elicits strong T-cell stimulation in a tumor-localized manner. Clin Cancer Res. (2022) 28:3387–99. doi: 10.1158/1078-0432.CCR-21-2762, PMID: 35121624 PMC9662934

[249] Warmuth S Gunde T Snell D Brock M Weinert C Simonin A. Engineering of a trispecific tumor-targeted immunotherapy incorporating 4-1BB co-stimulation and PD-L1 blockade. Oncoimmunology. (2021) 10:2004661. doi: 10.1080/2162402X.2021.2004661, PMID: 35844969 PMC9278964

[250] Snell D Gunde T Warmuth S Chatterjee B Brock M Hess C. An engineered T-cell engager with selectivity for high mesothelin-expressing cells and activity in the presence of soluble mesothelin. Oncoimmunology. (2023) 12:2233401. doi: 10.1080/2162402X.2023.2233401, PMID: 37456982 PMC10339761

[251] Ye QN Zhu L Liang J Zhao DK Tian TY Fan YN. Orchestrating NK and T cells via tri-specific nano-antibodies for synergistic antitumor immunity. Nat Commun. (2024) 15:6211. doi: 10.1038/s41467-024-50474-y, PMID: 39043643 PMC11266419

[252] Groeneveldt C Kinderman P van den Wollenberg DJM van den Oever RL Middelburg J Mustafa DAM. Preconditioning of the tumor microenvironment with oncolytic reovirus converts CD3-bispecific antibody treatment into effective immunotherapy. J Immunother Cancer. (2020) 8:e001191. doi: 10.1136/jitc-2020-001191, PMID: 33082167 PMC7577070

[253] Fajardo CA Guedan S Rojas LA Moreno R Arias-Badia M de Sostoa J. Oncolytic adenoviral delivery of an EGFR-targeting T-cell engager improves antitumor efficacy. Cancer Res. (2017) 77:2052–63. doi: 10.1158/0008-5472.CAN-16-1708, PMID: 28143835

[254] Basnet S Santos JM Quixabeira DCA Clubb JHA Grönberg-Vähä-Koskela SAM Arias V. Oncolytic adenovirus coding for bispecific T cell engager against human MUC-1 potentiates T cell response against solid tumors. Mol Ther Oncolytics. (2022) 28:59–73. doi: 10.1016/j.omto.2022.12.007, PMID: 36699617 PMC9842968

[255] Freedman JD Hagel J Scott EM Psallidas I Gupta A Spiers L. Oncolytic adenovirus expressing bispecific antibody targets T-cell cytotoxicity in cancer biopsies. EMBO Mol Med. (2017) 9:1067–87. doi: 10.15252/emmm.201707567, PMID: 28634161 PMC5538299

[256] Song Z Liu P Zhang D Wang T Yue W Geng Y. *In vivo* expression of anti-CD19/CD3 BiTE by liver-targeted AAV for the treatment of B cell Malignancies. Blood Cancer J. (2024) 14:46. doi: 10.1038/s41408-024-01036-4, PMID: 38485926 PMC10940594

[257] Zhu H Zhang W Guo Q Fan R Luo G Liu Y. Engineered oncolytic virus expressing B7H3-targeting BiTE enhances antitumor T-cell immune response. J Immunother Cancer. (2024) 12:e009901. doi: 10.1136/jitc-2024-009901, PMID: 39615894 PMC11624812

[258] O’Riordan CR Lachapelle A Delgado C Parkes V Wadsworth SC Smith AE. PEGylation of adenovirus with retention of infectivity and protection from neutralizing antibody *in vitro* and *in vivo* . Hum Gene Ther. (1999) 10:1349–58. doi: 10.1089/10430349950018021, PMID: 10365665

[259] Hofherr SE Shashkova EV Weaver EA Khare R Barry MA. Modification of adenoviral vectors with polyethylene glycol modulates *in vivo* tissue tropism and gene expression. Mol Ther. (2008) 16:1276–82. doi: 10.1038/mt.2008.86, PMID: 18461056

[260] Croyle MA Chirmule N Zhang Y Wilson JM. PEGylation of E1-deleted adenovirus vectors allows significant gene expression on readministration to liver. Hum Gene Ther. (2002) 13:1887–900. doi: 10.1089/104303402760372972, PMID: 12396620

[261] Heidbuechel JPW Engeland CE. Oncolytic viruses encoding bispecific T cell engagers: a blueprint for emerging immunovirotherapies. J Hematol Oncol. (2021) 14:63. doi: 10.1186/s13045-021-01075-5, PMID: 33863363 PMC8052795

[262] Staflin K Zuch de Zafra CL Schutt LK Clark V Zhong F Hristopoulos M. Target arm affinities determine preclinical efficacy and safety of anti-HER2/CD3 bispecific antibody. JCI Insight. (2020) 5:e133757. doi: 10.1172/jci.insight.133757, PMID: 32271166 PMC7205277

[263] Trinklein ND Pham D Schellenberger U Buelow B Boudreau A Choudhry P. Efficient tumor killing and minimal cytokine release with novel T-cell agonist bispecific antibodies. MAbs. (2019) 11:639–52. doi: 10.1080/19420862.2019.1574521, PMID: 30698484 PMC6601548

[264] Dang K Castello G Clarke SC Li Y Balasubramani A Boudreau A. Attenuating CD3 affinity in a PSMAxCD3 bispecific antibody enables killing of prostate tumor cells with reduced cytokine release. J Immunother Cancer. (2021) 9:e002488. doi: 10.1136/jitc-2021-002488, PMID: 34088740 PMC8183203

[265] Liu H Saxena A Sidhu SS Wu D. Fc engineering for developing therapeutic bispecific antibodies and novel scaffolds. Front Immunol. (2017) 8:38. doi: 10.3389/fimmu.2017.00038, PMID: 28184223 PMC5266686

[266] Gaballa S Hou J Devata S Seok-Goo C Nair R Yoon D. Evaluation of AZD0486, a novel CD19xCD3 T-cell engager, in relapsed/refractory diffuse large B-cell lymphoma in an ongoing first-in-human phase 1 study: high complete responses seen in CAR-T-naive and CAR-T-exposed patients. Blood. (2024)144:868. doi: 10.1182/blood-2024-193681

[267] Abdelmotaleb O Schneider A Gassner C Märsch S Klein C. The impact of CD3 affinity-attenuation on T cell engaging bispecific antibodies: is it really that simple? Expert Opin Drug Discov. (2025) 20:943–9. doi: 10.1080/17460441.2025.2522088, PMID: 40549538

[268] Sun Y Zhou L Gu X Zhao J Bi J Pan L. Leveraging T cell co-stimulation for enhanced therapeutic efficacy of trispecific antibodies targeting prostate cancer. J Immunother Cancer. (2025) 13:e010140. doi: 10.1136/jitc-2024-010140, PMID: 40081942 PMC11907039

[269] Hu S Fu W Xu W Yang Y Cruz M Berezov SD. Four-in-one antibodies have superior cancer inhibitory activity against EGFR, HER2, HER3, and VEGF through disruption of HER/MET crosstalk. Cancer Res. (2015) 75:159–70. doi: 10.1158/0008-5472.CAN-14-1670, PMID: 25371409

[270] ClinicalTrials.gov [Internet]. Identifier NCT05192486, A study to evaluate the safety, tolerability, pharmacokinetics, and preliminary antitumor activity of GNC-038 in subjects with relapsed or refractory diffuse large B-cell lymphoma. Bethesda (MD): National Library of Medicine (US) (2022). Available online at: https://clinicaltrials.gov/study/NCT05192486.

[271] ClinicalTrials.gov [Internet]. Identifier NCT06239194, Dose escalation and dose expansion study of MDX2001 in patients with advanced solid tumors. Bethesda (MD): National Library of Medicine (US) (2024). Available online at: https://clinicaltrials.gov/study/NCT06239194.

[272] ClinicalTrials.gov [Internet]. Identifier NCT06088654, Phase 1/2 study of IPH6501 in patients with relapsed/refractory B-cell non-Hodgkin lymphoma. Bethesda (MD): National Library of Medicine (US) (2023). Available online at: https://clinicaltrials.gov/study/NCT06088654.

[273] Ai Z Wang B Song Y Cheng P Liu X Sun P. Prodrug-based bispecific antibodies for cancer therapy: advances and future directions. Front Immunol. (2025) 16:1523693. doi: 10.3389/fimmu.2025.1523693, PMID: 39911391 PMC11794264

[274] Miura D Kato Y Yasunaga M Kumagai I Asano R. Design of a prodrug bispecific antibody masked by a functional molecule for lymphocyte activation for cancer therapy. J Biol Eng. (2025) 19:45. doi: 10.1186/s13036-025-00517-9, PMID: 40375288 PMC12079947

[275] Kavanaugh WM. Antibody prodrugs for cancer. Expert Opin Biol Ther. (2020) 20:163–71. doi: 10.1080/14712598.2020.1699053, PMID: 31779489

[276] Sulea T Rohani N Baardsnes J Corbeil CR Deprez C Cepero-Donates Y. Structure-based engineering of pH-dependent antibody binding for selective targeting of solid-tumor microenvironment. MAbs. (2020) 12:1682866. doi: 10.1080/19420862.2019.1682866, PMID: 31777319 PMC6927761

[277] Klaus T Deshmukh S. pH-responsive antibodies for therapeutic applications. J BioMed Sci. (2021) 28:11. doi: 10.1186/s12929-021-00709-7, PMID: 33482842 PMC7821552

[278] Fan S Han H Yan Z Lu Y He B Zhang Q. Lipid-based nanoparticles for cancer immunotherapy. Med Rev (2021). (2023) 3:230–69. doi: 10.1515/mr-2023-0020, PMID: 37789955 PMC10542882

[279] Wu L Wang W Tian J Qi C Cai Z Yan W. Engineered mRNA-expressed bispecific antibody prevent intestinal cancer via lipid nanoparticle delivery. Bioengineered. (2021) 12:12383–93. doi: 10.1080/21655979.2021.2003666, PMID: 34895063 PMC8810065

[280] Huang C Duan X Wang J Tian Q Ren Y Chen K. Lipid nanoparticle delivery system for mRNA encoding B7H3-redirected bispecific antibody displays potent antitumor effects on Malignant tumors. Adv Sci (Weinh). (2023) 10:e2205532. doi: 10.1002/advs.202205532, PMID: 36403209 PMC9875623

[281] Stadler CR Ellinghaus U Fischer L Bähr-Mahmud H Rao M Lindemann C. Preclinical efficacy and pharmacokinetics of an RNA-encoded T cell-engaging bispecific antibody targeting human claudin 6. Sci Transl Med. (2024) 16:eadl2720. doi: 10.1126/scitranslmed.adl2720, PMID: 38776391

[282] Hu L Zhang S Sienkiewicz J Zhou H Berahovich R Sun J. HER2-CD3-fc bispecific antibody-encoding mRNA delivered by lipid nanoparticles suppresses HER2-positive tumor growth. Vaccines (Basel). (2024) 12:808. doi: 10.3390/vaccines12070808, PMID: 39066446 PMC11281407

[283] Pan Y Chen R Lv X Wang Y Zhang H. Targeted delivery of IFN-α-anti-GPC3 fusion protein via mRNA-LNP platform elicits potent anti-tumor immunity in hepatocellular carcinoma. Drug Deliv Transl Res. (2025). doi: 10.1007/s13346-025-01911-y, PMID: 40908442

[284] Hangiu O Navarro R Frago S Rubio-Pérez L Tapia-Galisteo A Díez-Alonso L. Effective cancer immunotherapy combining mRNA-encoded bispecific antibodies that induce polyclonal T cell engagement and PD-L1-dependent 4-1BB costimulation. Front Immunol. (2025) 15:1494206. doi: 10.3389/fimmu.2024.1494206, PMID: 39835115 PMC11743637

[285] Zhang Y Gu X Huang L Yang Y He J. Enhancing precision medicine: Bispecific antibody-mediated targeted delivery of lipid nanoparticles for potential cancer therapy. Int J Pharm. (2024) 654:123990. doi: 10.1016/j.ijpharm.2024.123990, PMID: 38467208

[286] Amabile A Phelan M Yu Z Silva P Marks A Morla-Folch J. Bispecific antibody targeting of lipid nanoparticles. bioRxiv. (2024) 22:2024. doi: 10.1101/2024.12.20.629467, PMID: 39763831 PMC11702604

[287] Dietmair B Humphries J Mercer TR Thurecht KJ Howard CB Cheetham SW. Targeted mRNA delivery with bispecific antibodies that tether LNPs to cell surface markers. Mol Ther Nucleic Acids. (2025) 36:102520. doi: 10.1016/j.omtn.2025.102520, PMID: 40235853 PMC11999258

